# PHB2 promotes colorectal cancer cell proliferation and tumorigenesis through NDUFS1-mediated oxidative phosphorylation

**DOI:** 10.1038/s41419-023-05575-9

**Published:** 2023-01-20

**Authors:** Lin Ren, Li Meng, Jing Gao, Mingdian Lu, Chengyu Guo, Yunyun Li, Ziye Rong, Yan Ye

**Affiliations:** 1 Department of Immunology, School of Basic Medical Sciences, Anhui Medical University, Hefei, China; 2 Department of Blood Transfusion, The First Affiliated Hospital of Anhui Medical University, Hefei, China; 3 Department of Blood Transfusion, Anhui Public Health Clinical Center, Hefei, China; 4 Department of General Surgery, The First Affiliated Hospital of Anhui Medical University, Hefei, China

**Keywords:** Rectal cancer, Colon cancer

## Abstract

The alteration of cellular energy metabolism is a hallmark of colorectal cancer (CRC). Accumulating evidence has suggested oxidative phosphorylation (OXPHOS) is upregulated to meet the demand for energy in tumor initiation and development. However, the role of OXPHOS and its regulatory mechanism in CRC tumorigenesis and progression remain unclear. Here, we reveal that Prohibitin 2 (PHB2) expression is elevated in precancerous adenomas and CRC, which promotes cell proliferation and tumorigenesis of CRC. Additionally, knockdown of PHB2 significantly reduces mitochondrial OXPHOS levels in CRC cells. Meanwhile, NADH:ubiquinone oxidoreductase core subunit S1 (NDUFS1), as a PHB2 binding partner, is screened and identified by co-immunoprecipitation and mass spectrometry. Furthermore, PHB2 directly interacts with NDUFS1 and they co-localize in mitochondria, which facilitates NDUFS1 binding to NADH:ubiquinone oxidoreductase core subunit V1 (NDUFV1), regulating the activity of complex I. Consistently, partial inhibition of complex I activity also abrogates the increased cell proliferation induced by overexpression of PHB2 in normal human intestinal epithelial cells and CRC cells. Collectively, these results indicate that increased PHB2 directly interacts with NDUFS1 to stabilize mitochondrial complex I and enhance its activity, leading to upregulated OXPHOS levels, thereby promoting cell proliferation and tumorigenesis of CRC. Our findings provide a new perspective for understanding CRC energy metabolism, as well as novel intervention strategies for CRC therapeutics.

## Introduction

Colorectal cancer (CRC) is a common malignant tumor of the digestive system with high morbidity and mortality in the world [1, 2]. In most cases of CRC, normal intestinal mucosal hyperplasia forms benign adenomatous polyps (APs), which gradually grow and become malignant, and eventually lead to CRC infiltration and metastasis [3]. The prognosis of CRC is closely related to its stages, and metastatic CRC has become the leading cause of cancer-related death [4]. Thus, early detection and removal of polyps is considered to be effective in preventing CRC occurrence [5]. Although extensive studies have revealed that the activation of oncogene *KRAS* and the inactivation of some tumor suppressor genes, such as *APC*, *TP53*, and *18q* LOH, are involved in the pathological process of CRC [6–9], the bioenergetic alterations associated with this process are still obscure [10].

Decades ago, Otto Warburg found the glycolysis level of tumor cells was higher than that of normal cells, leading to the assumption of mitochondrial damage and downregulation of oxidative phosphorylation (OXPHOS) in tumor cells [11]. However, recent experimental evidence has suggested that mitochondrial respiratory function is intact in most tumor cells, and OXPHOS activity can also be upregulated in certain cancers and plays a vital role in cell proliferation, tumor metastasis, and drug resistance [12–14]. So far, the role of OXPHOS in CRC tumorigenesis and progression remains controversial. Wey-Ran Lin et al. demonstrated oxygen consumption rate (OCR) reduction and OXPHOS deficiency in CRC [15]. The opposite opinion pointed out that CRCs are not purely glycolytic tumors. The OXPHOS system is the substantial provider of ATP in these malignant cells, and inhibition of the OXPHOS level can suppress CRC proliferation and metastasis [16, 17]. Moreover, OXPHOS inhibitors have been found to be effective in targeting OXPHOS-upregulated cancer subtypes [12, 18, 19]. Although it has been reported that somatic mutation or increased amount of mitochondrial DNA (mtDNA) is correlated with the changes of OXPHOS levels in various tumors [12, 20, 21], Wey-Ran Lin et al. showed somatic mtDNA mutations accumulated in APs and CRC but were not associated with bioenergetic changes [15], indicating that other factors modulating OXPHOS levels may be involved.

Prohibitin 2 (PHB2) is a highly conserved and ubiquitous protein, which is found to be present in the nucleus, cytoplasm, plasma membrane, and mitochondria. It is involved in various cellular processes including transcription, signal transduction, and cellular metabolism [22], and is implicated in a variety of pathologies, such as metabolic diseases, neurodegeneration, and cancer [23, 24]. It has been reported that commonly up-regulated PHB2 has been found in certain tumors, such as esophageal squamous cell carcinoma (ESCC), non-small cell lung cancer (NSCLC), and hepatocellular carcinoma, and PHB2 is considered as a prognostic marker for ESCC and NSCLC [25–27]. PHB2 in different subcellular localizations has distinct roles in tumorigenesis and progression, such as the pro-tumorigenic effect of PHB2 in the cytoplasm of NSCLC cells and plasma membrane of rhabdomyosarcoma (RMS) cells [26, 28]. On the contrary, the nuclear translocation of PHB2 inhibits the proliferation and metastasis of ER-positive breast cancer cells [29]. These findings indicated the pathological roles of PHB2 outside the mitochondria in tumor development and progression. However, how PHB2 in the mitochondria affects this pathological process has not been elucidated. Of note, PHB2 forms a circular complex with PHB1 located in the mitochondrial inner membrane to maintain the stability of mitochondrial structure and functions including the assembly and activity of OXPHOS system complexes [22, 24]. In fact, PHB2 not only functions as a heterodimer complex but also performs its distinctive function independent of PHB1 [22, 30]. In view of the vital role of PHB2 in the regulation of mitochondrial respiration, and the increasing demand for energy in tumors, the altered expression of mitochondrial PHB2 in certain tumors should be involved in tumorigenesis and progression via regulating the cellular energy metabolism [22]. However, it remains unclear how PHB2 modulates bioenergetic profiles in tumors. Until now, there are few reports about the expression and biological functions of PHB2 in CRC. Therefore, whether mitochondrial PHB2 is involved in the development and progression of CRC, as well as the underlying mechanisms, is still worth to be explored.

In this study, we demonstrate for the first time that PHB2 expression is elevated in precancerous lesions and CRC, which promotes cell proliferation and tumorigenesis of CRC. Mechanistically, increased PHB2 directly interacts with NADH:ubiquinone oxidoreductase core subunit S1 (NDUFS1) to stabilize complex I, thereby enhancing OXPHOS levels and promoting cell proliferation in colorectal tumorigenesis and progression.

## Materials and methods

### Clinical specimens

The human colorectal cancer precancerous tissues, colorectal cancer tissues, and normal adjacent tissues were collected from the First Affiliated Hospital of Anhui Medical University in this study. The clinical characteristics of the patients are shown in Supplementary Table S2. Studies using human tissues were approved by the Ethics Committee of Anhui Medical University.

### Cell culture

The human CRC cell lines were obtained from the Cell Bank of the Chinese Academy of Sciences (Shanghai, China). All cells were cultured in Dulbecco’s modified Eagle’s medium (DMEM) supplemented with 10% fetal bovine serum (FBS) (Gibco, Grand Island, USA), 100 U/mL penicillin and 100 μg/mL streptomycin at 37 °C in a 5% CO_2_ humid environment. All cell lines used in this study were authenticated by STR profiling and tested for mycoplasma contamination.

### Immunohistochemistry (IHC)

IHC analysis was carried out as previously described [31]. Briefly, after the patient’s tissue was removed, samples were fixed within formalin for at least 12 h, embedded in paraffin, and sliced into 4-μm-thick sections. After antigen retrieval, sections were incubated overnight at 4 °C in anti-PHB2 (1:100, Proteintech, Chicago, USA) or anti-Ki67 (1:100, Proteintech), overnight at 4 °C, respectively. Sections were then detected with the Dako Envision HRP Detection system/DAB (Dako, Heverlee, Belgium) according to the manufacturer’s instructions. IHC staining was evaluated by two independent pathologists using a previously described semiquantitative scoring system [31]. The staining intensity was scored as 1 (negative), 2 (weak), 3 (moderate), and 4 (intense). The percentage of positive cells was quantified as 0 (≤5%), 1 (6–25%), 2 (26–50%), 3 (51–75%), and 4 (>75%). For statistical analysis, the sum score (immunoreactivity score (IRS)) of intensity and extent of staining was calculated. The final score of ≤4 was considered to be low and the score of >4 was considered to be high.

### Plasmids and lentiviruses

The full-length cDNA of PHB2 and NDUFS1 was amplified and sub-cloned into the p3xFlag-Myc-CMV24 and pcDNA-HA vectors (Hanheng Biotechnology, Shanghai, China), respectively. Cells were transfected with 2 μg plasmid or the empty vector in Opti-MEM medium (Gibco) using Lipofectamine 2000 reagent (Invitrogen, Carlsbad, CA, USA) according to the manufacturer’s instructions.

For lentivirus-mediated PHB2 short hairpin RNA (shRNA) silencing, predesigned shRNA sequences (PHB2-shRNA #1 Forward: 5’-TGAGCTTTAGCCGAGAGTA-3’ and Reverse: 5’-TACTCTCGGCTAAAGCTCA-3’; PHB2-shRNA #2 Forward: 5’-GAGCAAGAACCCTGGCTACAT-3’ and Reverse: 5’-ATGTAGCCAGGGTTCTTGCTC-3’) were cloned into the lentiviral vector GV248 (GeneChem, Shanghai, China). Lentiviral infection of cells was performed according to the manufacturer’s protocols. Briefly, HCT116 and HT-29 cells were infected with the lentivirus for 72 h and then screened with puromycin (2 ng/mL) for 2 weeks. The cells were then cultured for further research.

### siRNA transfection

siRNA targeting NDUFS1 and negative control siRNA were purchased from GenePharma (Shanghai, China). The target gene sequences of siRNA-NDUFS1 were as follows: si-NDUFS1 Forward: 5’-GCACAGAUUUGCGUUCCAAUU-3’ and Reverse: 5’-AAUUGGAACGCAAAUCUGUGC-3’. According to the manufacturer’s protocol, siRNA duplexes were transfected into cells in an opti-MEM medium using Lipofectamine 2000 reagent.

### Western blot

Western blot was carried out as described previously [32]. The specific primary antibodies against PHB2 (1:2000), β-actin (1:5000), Flag tag (1:1000), NDUFS1 (1:2000), HA tag (1:4000), GST tag (1:5000), and NDUFV1 (1:1000) were purchased from Proteintech Group. Immunoreactive bands were revealed with the ECL Advance Western Blotting Detection Kit (Amersham Bioscience) and visualized by Tanon 4500SF image system (Tanon, Shanghai, China).

### RNA isolation and real-time quantitative PCR analysis (qRT-PCR)

RNA isolation and qRT-PCR were carried out as described previously [32]. GAPDH was used as a reference gene and results were normalized to GAPDH expression. Detailed sequences for each primer pair were as follows: PHB2 Forward: 5’-CAGAGCTGAGCTTTAGCCGA-3’ and Reverse: 5’-CTGCACAATTTTCTGCCGCT-3’; GAPDH Forward: 5’-GGACCTGACCTGCCGTCTAG-3’ and Reverse: 5’-GTAGCCCAGGATGCCCTTGA-3’.

### Quantification of mtDNA

Total DNA was extracted from HCT116 and HT-29 cells using the Genomic DNA Purification Kit (Beyotime, China), according to the manufacturer’s protocol. mtDNA content was quantified by real-time quantitative PCR. Briefly, the ND1 gene and the β-actin gene were used to quantify the copy number of mtDNA and nuclear DNA (nDNA), respectively. The primers for the mtDNA were ND1 Forward: 5’-CCCTAAAACCCGCCACATCT-3’ and Reverse: 5’-TAGAAGAGCGATGGTGAGAGCTAA-3’. The primers for nDNA were β-actin Forward: 5’-CCCAGCCATGTACGTTGCTA-3’ and Reverse: 5’-CGTCACCGGAGTCCATCAC-3’. mtDNA content was expressed as the mtDNA:nDNA ratio.

### Cell viability

Cell viability was evaluated by 3-(4,5-dimethylthiazol-2-yl)-2,5-diphenyltetrazolium bromide (MTT) assay. Cells were plated at a density of 3 000 cells in 96-well plates overnight before treatment as desired. Twenty μL MTT (5 mg/mL, Beyotime) was added at different time points and incubated for 4 h. After removing the medium, 150 μL DMSO was added and incubated at 37 °C for 10 min, and the absorbance was measured at 490 nm using a universal microplate reader (Bio-Tek, Winooski, USA).

Cells transfected with Flag-PHB2 plasmid were seeded in 96-well plates (8 × 10^3^ cells/well), and then treated with complex I inhibitor rotenone or IACS-10759 (Topscience, Shanghai, China) at the corresponding concentration for 24 h, then 20 μL MTT was added to detect cell viability.

### BrdU incorporation assays

Cell proliferation was evaluated using BrdU Cell Proliferation Assay Kit (Cell Signaling, Beverly, USA) as per the manufacturer’s instructions. Briefly, cells were seeded at 8 × 10^3^/well on 96-well plates and cultured overnight. Ten μL BrdU (10 mM) was added and incubated for 24 h before BrdU assays were carried out. Absorbance was read at 450 nm.

### Colony formation assays and soft agar colony formation assays

For the colony formation assays, 500 cells per well were plated in six-well culture plates and cultured for 12–14 days at 37 °C in the presence of 5% CO_2_, then fixed with methanol and stained with crystal violet (0.5% solution, Beyotime).

For the soft agar colony formation assays, 2 × 10^3^ cells/well were suspended in a culture medium containing 0.6% low-melting point agarose (Sigma-Aldrich), which was on top of 1.2% agarose bottom layer in 6-well plates. Cells were then incubated for a further 21 days and stained with MTT.

### Co-immunoprecipitation assays (co-IP)

Co-IP assays were conducted as previously described [32]. The cell lysates (1 mg total protein) were incubated with the corresponding target antibodies (3 µg) at 4 °C overnight. Then, protein A/G agarose beads (Invitrogen) were added and incubated at 4 °C for 4 h. After washing the beads, the samples were analyzed by western blot.

### Immunofluorescence staining

Immunofluorescence staining was conducted as previously described [32]. Cells were incubated with anti-PHB2 mouse antibodies (1:50, Proteintech) and anti-NDUFS1 rabbit antibodies (1:50, Proteintech) overnight at 4 °C. Then, Cells were incubated at room temperature with Alexa Flour 488 anti-rabbit IgG antibodies (1:100, Proteintech) and Alexa Flour 405 anti-mouse IgG antibodies (1:100, Proteintech) for 1 h protected from light. The co-localization of PHB2 and NDUFS1 was detected by a confocal laser scanning microscope (Carl Zeiss, Oberkochene, Germany).

### MitoTracker staining

Cells were incubated with 200 nM MitoTracker Red CMXRos probe (Beyotime) at room temperature for 30 min to label mitochondria. Images were obtained with a confocal laser scanning microscope (Carl Zeiss).

### Detection of mitochondrial ROS (mtROS)

Mitochondrial superoxide level was determined using the fluorescent probe MitoSOX™ (Invitrogen). Briefly, 1 mL MitoSOX™ (5 μM) was added and incubated for 10 min at 37 °C in the dark. The mtROS levels were measured by confocal laser scanning microscope (Carl Zeiss) or flow cytometry (BD FACSverse3, New Jersey, USA).

### Colorectal xenograft mouse model

Viable HT-29.shPHB2 and HT-29.NC cells (6 × 10^6^ cells per mouse) were injected subcutaneously into the right posterior flanks of nu/nu mice (4–5-weeks old, female, *n* = 6/group, Gem Pharmatech, Jiangsu, China). Tumor volume was measured every 3 days for a total of 24 days. Tumor volume was calculated as 1/2 × tumor length (mm) × tumor width^2^ (mm^2^). Then, the mice were sacrificed and tumors were measured and weighed. Animal studies were approved by the Animal Research Ethics Committee of the Anhui Medical University of China.

### IP-mass spectrometry

The lysates of HCT116 cells were immunoprecipitated with anti-PHB2 (4 μg, Proteintech) antibody or control IgG antibody (4 μg, Proteintech), separated by electrophoresis, and finally visualized with Coomassie brilliant blue staining. The gel strips were excised and sent to Shanghai Applied Protein Technology (Shanghai, China) for LC-MS/MS analysis.

### GST-pull down assay

After induction of expression, purification of soluble GST-tagged proteins was performed using GST label protein purification kits (Beyotime) according to the manufacturer’s instructions. PHB2 cDNA was amplified and cloned into a pGEX-5X-3 vector and expressed as glutathione-s-transferase (GST) fusion proteins in *E. coli* BL21 (DE3) (TSINGKE Biological, Nanjing, China). For the GST pull-down assay, HCT116 and HT-29 cell lysates were incubated with purified GST fusion proteins at 4 °C for 4 h. Then GST-Tag Purification Resin was used to precipitate GST tag proteins and their binding protein. Finally, the resin was suspended with elution buffer and centrifuged at 1000 × *g* for 10 s. The supernatant was collected for subsequent western blot analysis.

### Intracellular ATP level assay

Intracellular ATP level was measured using ATP Assay Kits (Beyotime) according to the manufacturer’s instructions. The relative luminescence unit (RLU) was measured by a Single-tube luminometer (Promega, Madison, USA).

### Mitochondrial complex I activity assay

Mitochondrial complex I activity was measured using a Mitochondrial Complex I Activity Assay Kit (Solarbio) following the manufacturer’s instructions. Complex I in mitochondria was extracted from 5 × 10^6^ cells, and the enzyme activity of complex I was assessed by measuring nicotinamide adenine dinucleotide (NADH) oxidation over 2 min at 340 nm.

### Mitochondrial oxygen consumption rate assay

Mitochondrial oxygen consumption rate (OCR) was measured with a XF96 Seahorse extracellular flux analyzer (Agilent Seahorse Bioscience, Santa Clara, CA, USA). Briefly, HCT116 and HT-29 cells with or without shPHB2 were inoculated to Seahorse XF96 cell culture microplate at a density of 25,000 cells per well overnight. A sensor probe plate was hydrated overnight with Seahorse XF calibration solution in a CO_2_-free incubator at 37 °C. One hour before measurements, the cell growth medium in the microplate was replaced with 180 μL XF assay medium (DMEM supplemented with 10 mM glucose, 2 mM glutamine, and 1 mM pyruvate, pH 7.4), and then the microplate was placed in a CO_2_-free incubator at 37 °C.

Stock solutions of oligomycin (1 μM), trifluoromethoxy carbonyl cyanide phenylhydrazone (FCCP, 1.25 μM), and rotenone (0.5 μM)/antimycin A (0.5 μM) in an XF Cell Mito Stress Test Kit were prepared in XF assay medium and added into the probe plates A, B, and C ports, respectively. Measurements were obtained at 37 °C. Data were collected using *Wave* 2.2.0.276 software (Agilent).

### Statistical analysis

Statistical analysis was performed using GraphPad Prism 8.3.0 (GraphPad Software, San Diego, CA, USA). Two-sided paired *t* tests were used to compare the differences between the two groups. All quantitative data were expressed as mean ± SEM, and the data were obtained from at least three independent experiments. *p < 0.05 was regarded as statistically significant.

## Results

### PHB2 expression is frequently upregulated in precancerous lesions and tumor tissues of human CRC

**Fig. 1:**
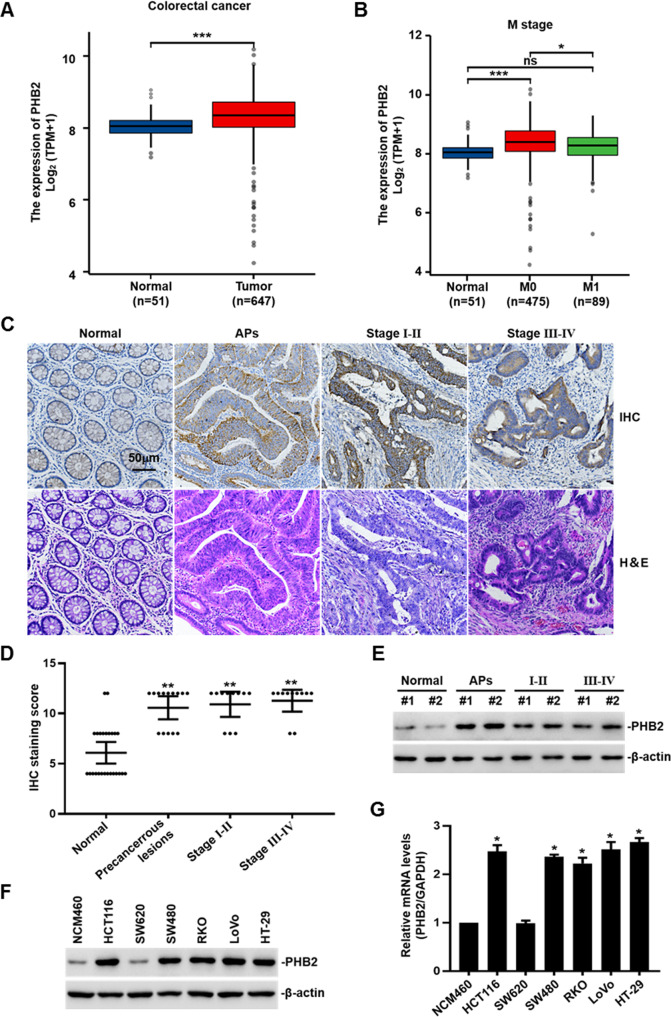
PHB2 expression is frequently upregulated in precancerous lesions and tumor tissues of human CRC. **A**, **B** The expression of PHB2 in CRC and adjacent tissues was analyzed by the TCGA database. **C** Representative microphotographs of IHC and H&E staining of PHB2 (brown) on adjacent tissues (*n* = 26), precancerous lesions (*n* = 14), and CRC stage I–II (*n* = 11) and stage III–IV (*n* = 11) tissue sections. **D** Quantification analysis of PHB2 expression in precancerous lesions, CRC, and adjacent tissues. **E** The PHB2 expression levels in tissues of colorectal adenomatous polyps (APs) (*n* = 2), CRC (*n* = 4), and control patients (*n* = 2) were detected by western blot. **F** Whole-cell lysates from a panel of CRC cells and normal intestinal epithelial cells were subjected to western blot. **G** Total RNA from a panel of CRC cells and normal intestinal epithelial cells were subjected to qRT-PCR. The relative abundance of PHB2 mRNA expression in NCM460 was arbitrarily designated as 1 (*n* = 3). Mean ± SEM, *p < 0.05, **p < 0.01, ***p < 0.001.

To assess the functions of PHB2 in CRC, we first investigated PHB2 expression in colorectal adenocarcinoma samples by analyzing the TCGA database (https://portal.gdc.cancer.gov/). The analyzed results showed that PHB2 was significantly upregulated in CRC compared with normal adjacent tissues (Fig. 1A). Furthermore, we analyzed PHB2 expression in CRC tissues with different stages and found that significantly increased PHB2 expression was only in the primary CRC samples (M0 stage without lymph node and distant metastasis) (Fig. 1B). These results suggested altered PHB2 expression occurs in earlier stages of CRC tumorigenesis. Next, we determined PHB2 protein levels in precancerous lesions, colorectal adenocarcinoma tissues with different stages, and adjacent normal tissues by immunohistochemistry (IHC) and western blot analysis. The results showed that compared to adjacent normal tissues, PHB2 expression was significantly increased in the precancerous lesions tissues, as well as primary and metastatic CRC tissues (Fig. 1C–E). Meanwhile, we examined PHB2 expression in a panel of CRC cell lines and normal human intestinal epithelial cells NCM460 by use of western blot and qRT-PCR analysis, and found that PHB2 expression in CRC cells was frequently higher than that of NCM460 cells (Fig. 1F, G). Our results above demonstrated that PHB2 expression levels were elevated in precancerous lesions and CRC, indicating it might be related to tumorigenesis and progression of CRC.

### PHB2 promotes cell proliferation and tumorigenesis of CRC

**Fig. 2:**
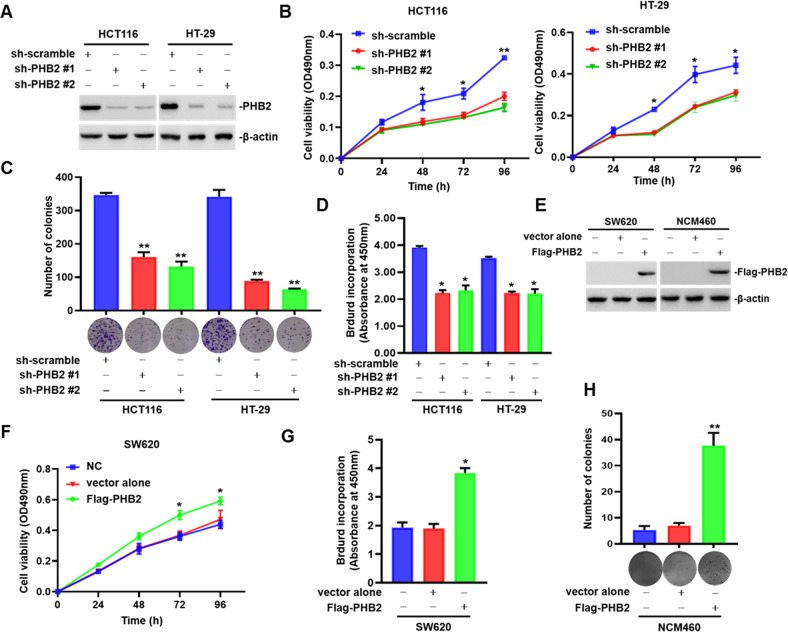
PHB2 promotes cell proliferation of CRC and the malignant transformation ability of normal human intestinal epithelial cells. Lentivirus encoding negative control shRNA or PHB2 shRNA was transduced into HCT116 and HT-29 cells. **A** Western blot analysis of the whole cell lysates. **B** Cell viability was measured by MTT assays. **C** Representative images and quantitation of clonogenic assays. **D** Cell proliferation was detected by BrdU incorporation assays. **E** SW620 and NCM460 cells were transiently transfected with empty vector or Flag-PHB2 plasmids for 48 h. The whole cell lysates were subjected to western blot. **F**, **G** Cell viability and proliferation of SW620 cells transfected with Flag-PHB2 plasmids were measured by MTT assays (**F**) and BrdU incorporation assays (**G**). **H** Representative images and quantitation of anchorage-independent growth of NCM460 cells transfected with Flag-PHB2 plasmids. Mean ± SEM, *p < 0.05, **p < 0.01.

To further determine the role of PHB2 in CRC development and progression, we first knocked down PHB2 in HCT116 and HT-29 cells with relatively high PHB2 expression by transducing lentivirus-mediated PHB2 shRNA and detected the effect of PHB2 on cell proliferation of CRC. The results showed that the knockdown of PHB2 significantly inhibited CRC cell proliferation (Fig. 2A–D). Moreover, we overexpressed PHB2 in SW620 cells with relatively low expression of PHB2 and also found that PHB2 promoted CRC cell proliferation by MTT and BrdU incorporation assays (Fig. 2E–G). Because of increased PHB2 expression in precancerous lesions of CRC, we performed soft agar colony formation assays to explore whether PHB2 promotes the malignant transformation of normal human intestinal epithelial cells. As shown in Fig. 2H, overexpression of PHB2 promoted anchor-independent growth of NCM460 cells, suggesting that PHB2 could enhance the malignant transformation ability of normal human intestinal epithelial cells.

**Fig. 3:**
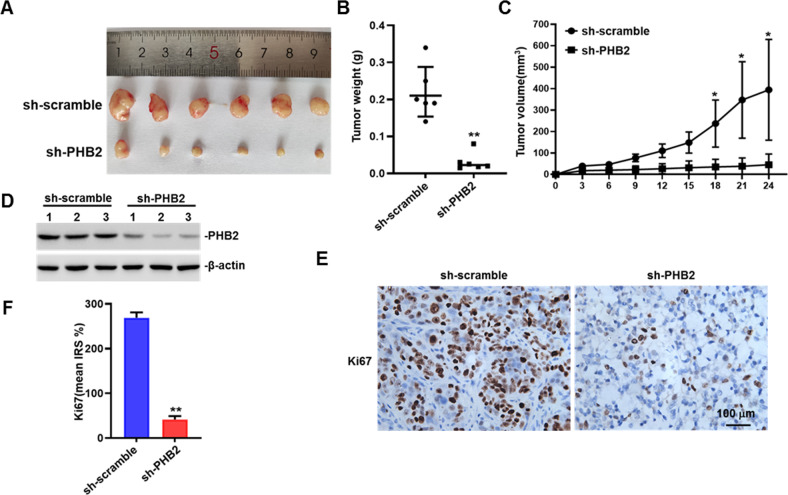
Knockdown of PHB2 retards CRC growth in a xenograft mouse model. **A** Representative images of xenograft tumors. **B** Comparison of tumors weights in animals implanted with HT-29.sh-scramble and HT-29.sh-PHB2 cells (*n* = 6). **C** The growth curves of xenograft tumors are presented. Tumor volume was calculated with a modified ellipsoidal formula (*n* = 6). **D** The whole-cell lysates of crude tumor tissues from three randomly sampled tumors from each group were subject to western blot. **E** Representative images of IHC staining with anti-Ki67 in xenograft tumors. **F** Quantification analysis of Ki67 expression in xenograft tumors. Mean ± SEM, *p < 0.05, **p < 0.01.

In the xenograft mouse model using HT-29 cells, knockdown of PHB2 retarded the tumor growth of CRC (Fig. 3A–C). Subsequently, we detected the expression of PHB2 and Ki67 in tumor tissue sections by western blot and IHC assays, respectively. The results showed that PHB2 and Ki67 expression was both decreased in the PHB2-knockdown group compared with the control group (Fig. 3D, E). Collectively, these results revealed that PHB2 promoted cell proliferation and tumorigenesis of CRC.

### Knockdown of PHB2 reduces mitochondrial OXPHOS level in CRC cells

**Fig. 4:**
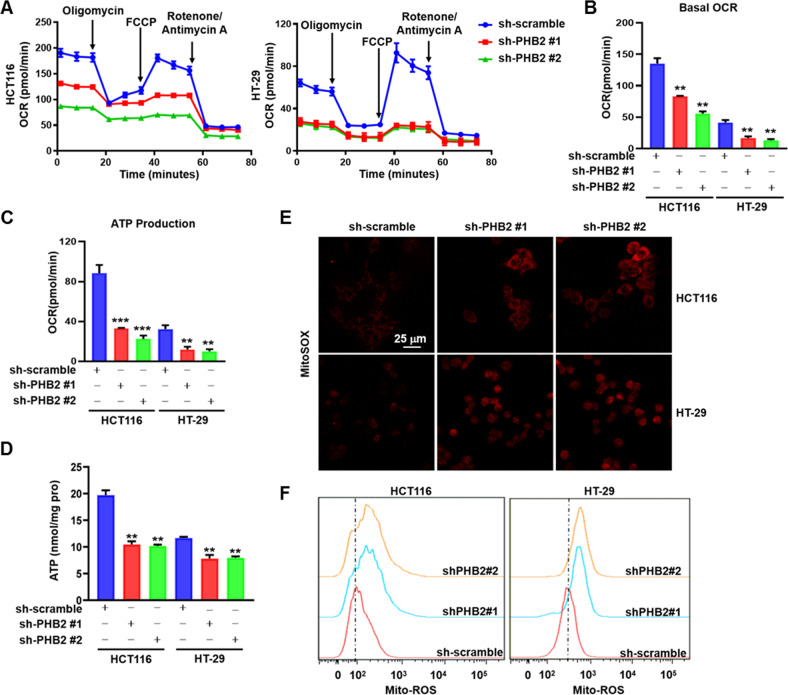
Knockdown of PHB2 reduces mitochondrial OXPHOS in CRC cells. **A** Mitochondrial OXPHOS activity in control, PHB2-deficient HCT116 and HT-29 cells were measured by Seahorse Bioscience XF96 extracellular flux analyzer. After establishing a baseline, oligomycin (1 μM), FCCP (1.5 μM), rotenone (0.5 μM), and antimycin A (0.5 μM) were sequentially added, as indicated by arrows. **B**, **C** Quantification of basal respiration and ATP production. **D** ATP content of HCT116 and HT-29 cells infected with shRNA against PHB2 was indicated by absorbance change measured at 340 nm. **E**, **F** Mitochondrial ROS levels were analyzed by confocal microscopy (**E**) and flow cytometry (**F**) after staining with MitoSOX™ (5 μM). Mean ± SEM, **p < 0.01, ***p < 0.001.

Given that mitochondrial OXPHOS plays an important role in cell proliferation and metastasis of certain tumors [13], in order to understand whether increased PHB2 expression promotes CRC tumorigenesis and progression by upregulating OXPHOS level, we first performed Seahorse MitoStress analysis to evaluate the effect of PHB2 knockdown on OXPHOS level of CRC cells. As shown in Fig. 4A, knockdown of PHB2 altered the response of CRC cells to mitochondrial electron transport chain (ETC) complexes inhibitors (including Oligomycin, an ATP synthase (complex V) inhibitor; Carbonyl cyanide-4 (trifluoromethoxy) Phenylhydrazone (FCCP), a mitochondrial uncoupler; rotenone, a complex I inhibitor; and Antimycin A, a complex III inhibitor), indicating a significant decrease of OXPHOS level (Fig. 4A). Further analysis of the above data showed that basal respiration and ATP production in the PHB2-knockdown CRC cells were significantly lower than those in the control CRC cells (Fig. 4B, C). We also measured glycolysis level after depletion of PHB2 in CRC cells, and found that extracellular acidification rate (ECAR) had no significant change (Supplementary Fig. S1A, B), which was consistent with the result of the lactate production detection (Supplementary Fig. S1C). Additionally, we detected the intracellular ATP level in PHB2-knockdown CRC cells, and found there was a significant reduction of the ATP level after PHB2 depletion (Fig. 4D). Mitochondrial OXPHOS dysfunction generally results in altered mitochondrial reactive oxygen species (ROS) levels [33]. Therefore, we examined the effect of PHB2 knockdown on mitochondrial ROS (mtROS) production. Indeed, our data showed that the knockdown of PHB2 led to increased mtROS production (Fig. 4E, F). Together, these data suggested that upregulated PHB2 promoted OXPHOS in CRC cells, which is critical for cell proliferation and tumorigenesis of CRC.

### PHB2 directly interacts with NDUFS1 to regulate complex I activity in the mitochondria of CRC cells

**Fig. 5:**
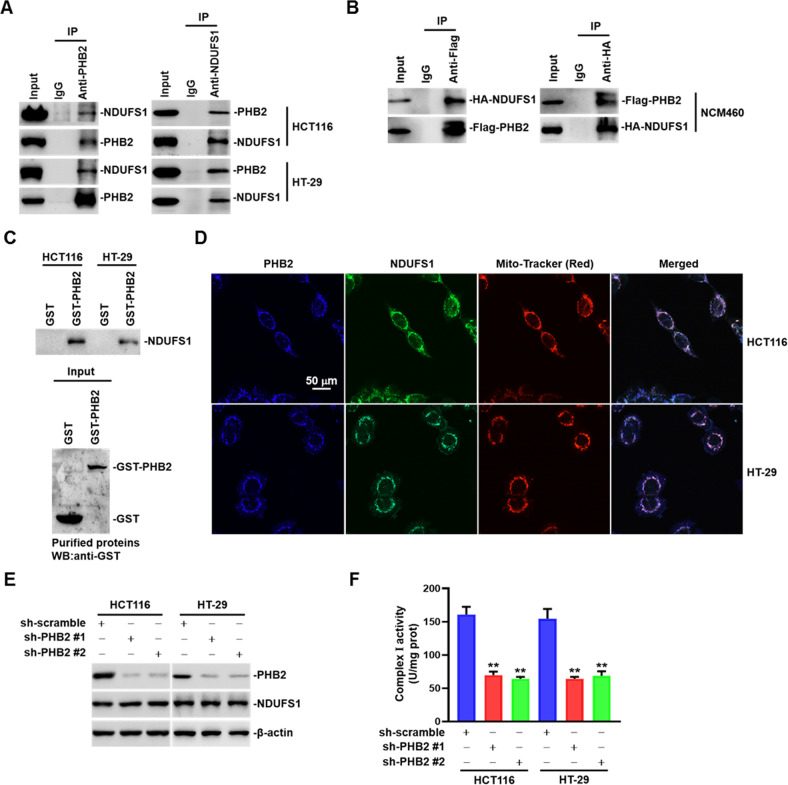
PHB2 directly interacts with NDUFS1 to regulate complex I activity in the mitochondria of CRC cells. **A** Whole-cell lysates from HCT116 and HT-29 cells were subjected to immunoprecipitation with anti-PHB2 and anti-NDUFS1 antibodies. The Immunoglobulin G (IgG) was used as a negative control. **B** Whole-cell lysates from NCM460 cells co-transfected with Flag-PHB2 and HA-NDUFS1 plasmids were subjected to anti-Flag and anti-HA immunoprecipitation, respectively. **C** Top: Recombinant GST-PHB2 pulls down NDUFS1 from the cell lysates of HCT116 and HT-29 cells. Bottom: Western blot analysis of purified recombinant GST proteins from a pull-down assay. **D** Representative immunofluorescence images showing the co-localization of PHB2 (blue), NDUFS1 (green) and mitochondria (red) in HCT116 and HT-29 cells. **E** Western blot analysis of the whole cell lysates. **F** Analysis of complex I activity in HCT116 and HT-29 cells infected with shRNA against PHB2. Mean ± SEM, **p < 0.01.

To further investigate the mechanisms of upregulated OXPHOS by PHB2, we screened the potential interacting proteins of PHB2 in HCT116 cells by immunoprecipitation and mass spectrometry analysis. Considering the main localization and vital functions of PHB2 in mitochondria, we focused on the PHB2-interacting proteins which are in mitochondria and associated with mitochondrial OXPHOS, among which the mitochondrial complex I subunit NDUFS1 attracted our attention (Supplementary Table S1). Next, we chose HT-29 and HT116 cells to identify the interaction between PHB2 and NDUFS1 by co-immunoprecipitation (co-IP) assays, and the results showed an endogenous interaction between PHB2 and NDUFS1 in CRC cells (Fig. 5A). Furthermore, we performed co-IP assays to verify the exogenous interaction by co-transfecting the Flag-PHB2 and HA-NDUFS1 plasmids into NCM460 cells. As shown in Fig. 5B, PHB2 and NDUFS1 reciprocally immunoprecipitated each other. It is well known that mitochondrial complex I is a huge complex formed by the close binding of multiple subunits, and it has been reported that PHB2 may bind to some of the complex I subunits [34]. However, these interactions have not been experimentally verified. To determine whether PHB2 binds NDUFS1 directly, the results of GST pull-down assays showed that purified GST-PHB2 pulled down NDUFS1 in HCT116 and HT-29 cell lysates (Fig. 5C). In addition, we used a Mito-tracker to detect the co-localization of PHB2 and NDUFS1, and found they were co-localized primarily in the mitochondria of CRC cells (Fig. 5D).

To further explore the effect of the interaction between PHB2 and NDUFS1, we first detect whether this interaction resulted in the altered protein expression of NDUFS1, and found that knockdown of PHB2 did not cause the significant changes of NDUFS1 protein expression levels (Fig. 5E). Since NDUFS1 is the core subunit of mitochondrial complex I and is involved in regulating OXPHOS levels, we then examined complex I activity in PHB2-knockdown CRC cells. The results showed that the mitochondrial complex I activity was significantly decreased after PHB2 depletion (Fig. 5F). In addition, we determined other multimeric enzymes activities of electron transport chain (ETC) complexes in PHB2-knockdown CRC cells, as shown in Supplementary Fig. S2, the activities of ubiquinone-cytochrome c oxidoreductase (complex III) and cytochrome c oxidase (complex IV) were subsequently reduced, while the activity of succinate dehydrogenase (complex II) had no significant change. Taken together, these results suggested that PHB2 interacted directly with NDUFS1 to regulate complex I activity in the mitochondrial of CRC cells.

### PHB2 promotes OXPHOS and cell proliferation through stabilizing mitochondrial complex I in CRC cells

**Fig. 6:**
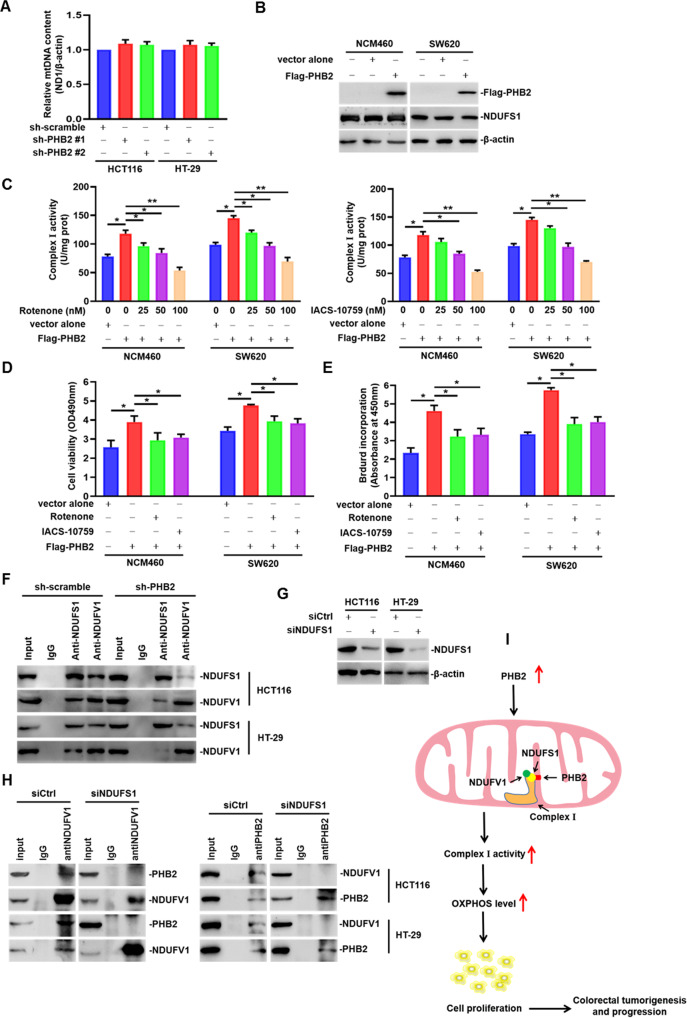
PHB2 promotes OXPHOS and cell proliferation through stabilizing mitochondrial complex I in CRC cells. **A** Mitochondrial DNA (mtDNA) content was measured by qPCR. **B** Western blot analysis of the whole cell lysates from NCM460 and SW620 cells transfected with Flag-PHB2 plasmids. **C** NCM460 and SW620 cells were transfected with Flag-PHB2 plasmids for 48 h, and then the cells were treated with rotenone at concentrations of 0, 25, 50, 100 nM, or IACS-10759 at concentrations of 0, 30, 60, 120 nM for 24 h. Complex I activity was measured by a microplate reader displaying absorbance at 340 nm. Empty vector transfection served as control. **D**, **E** NCM460 and SW620 cells overexpressed PHB2 were treated with rotenone (50 nM) or IACS-10759 (60 nM) for 24 h. Cell viability and proliferation were measured by MTT assays (**D**) and BrdU incorporation assays (**E**). **F** HCT116 and HT-29 cells infected with shRNA against PHB2 were subjected to immunoprecipitation with anti-NDUFS1 or anti-NDUFV1. **G**, **H** HCT116 and HT-29 cells were transfected with siRNA against NDUFS1. Whole cell lysates were subjected to western blot (**G**) and immunoprecipitation with anti-PHB2 or anti-NDUFV1 (**H**). **I** Schematic illustration of the potential mechanism by which elevated PHB2 expression promotes cell proliferation and tumorigenesis of CRC. Mean ± SEM, *p < 0.05, **p < 0.01.

As mentioned before, the OXPHOS level regulated by PHB2 may be related to mtDNA contents. Then, we detected the mtDNA contents in PHB2-knockdown CRC cells by use of qPCR analysis, however, the results indicated there were no significant changes in mtDNA levels in PHB2-knockdown CRC cells compared with the control cells (Fig. 6A). In order to determine whether the effect of PHB2 on CRC cell proliferation is due to regulation of complex I activity, we used two complex I inhibitors, rotenone, and IACS-10759, to inhibit complex I activity in PHB2-overexpressing NCM460 and SW620 cells (Fig. 6B) and chose the concentrations of two inhibitors which inhibited elevated complex I activity to levels similar to the control cells as working concentrations for the subsequent experiments. As shown in Fig. 6C, we treated PHB2-overexpressing NCM460 and SW620 cells with rotenone at the concentration of 50 nM and IACS-10759 at the concentration of 60 nM for 24 h, and then performed MTT and BrdU incorporation assays. The results showed that partial inhibition of complex I activity could abrogate the increased cell proliferation induced by overexpression of PHB2 in NCM460 and SW620 cells (Fig. 6D, E).

As we know, in complex I, NDUFS1 is tightly bound to NADH:ubiquinone oxidoreductase core subunit V1 (NDUFV1). Therein, NADH is oxidized by a flavin mononucleotide in NDUFV1, and then electrons enter the respiratory chain and transfer along a chain of FeS clusters [35]. In the next, to further explore how complex I activity was regulated by NDUFS1-interacting PHB2, we conducted co-IP assays with anti-NDUFS1 or anti-NDUFV1 antibodies in the presence or absence of PHB2. The results showed that, compared to the control CRC cells, the binding of NDUFS1 and NDUFV1 was decreased in the PHB2-knockdown cells (Fig. 6F). To determine the binding patterns of the three proteins, we performed co-IP assays with anti-PHB2 or anti-NDUFV1 antibodies in the NDUFS1-knockdown CRC cells (Fig. 6G, H). As shown in Fig. 6H, the combination of PHB2 and NDUFV1 was not detected after knocking down NDUFS1, suggesting that PHB2 might bind indirectly to NDUFV1 by NDUFS1. These results indicated that the binding of PHB2 to NDUFS1 facilitated the interaction between NDUFS1 and NDUFV1, which promoted the activity of complex I. Collectively, it was proposed that upregulated PHB2 interacted with NDUFS1 to stabilize the structure of complex I and enhance its activity, leading to increased OXPHOS levels, thereby promoting cell proliferation and tumorigenesis of CRC (Fig. 6I).

## Discussion

CRC is one of the most common malignant tumors in the world and ranks second in cancer-related deaths [1]. Endoscopic screening has been shown to reduce CRC incidence and mortality. However, prognostication and precise targeted therapies in CRC patients will facilitate improving early detection and personalized medicine, while the consensus molecular subtypes (CMS) classification reflects the heterogeneity of CRC [36]. Here in this report, we demonstrate PHB2 as a bioenergetics metabolism-related scaffolding protein, which is upregulated in precancerous adenomas and CRC and promotes cell proliferation and tumorigenesis of CRC. We also identify NDUFS1, a complex I subunit, as a novel PHB2-interacting partner in mitochondria, which is involved in the regulation of OXPHOS by PHB2 in CRC tumorigenesis and progression. This study provides a new perspective for the comprehensive understanding of CRC molecular profiling, as well as novel intervention strategies for CRC therapeutics.

The conventional adenoma-carcinoma-metastasis model occurs in ~70% of CRCs, which is associated with metabolic alterations caused by “*APC*-*KRAS*-*TP53*” specific genetic alteration (Vogelstein model) [37], and metabolic reprogramming is essential for the initiation and progression of CRC. In this study, we demonstrated that PHB2, a mitochondrial inner membrane scaffolding protein, was continuously increased during the adenoma-carcinoma-metastasis sequence of CRC, which contributed to OXPHOS promotion, thereby leading to tumorigenesis and progression. Although our IHC results showed upregulated PHB2 in the precancerous adenoma, primary and metastatic stages of CRC, there was increased expression of PHB2 in primary CRC but not in metastatic CRC from the TCGA database analysis. This discrepancy is largely due to the samples from diverse sources. Furthermore, how PHB2 is upregulated in CRC remains unclear. It has been reported that the promoter regions of PHB2 contain multiple transcription factor binding sites, including GATA-1,-2,-3, FoxO (Forkhead box protein), STAT (signal transducer and activator of transcription) -1,-3,-5, C/EBP-α (CAAT-enhancer binding protein-α), NF-κB (nuclear factor kappa-B) and Sp1 [22]. Among them, constitutive transcription factors such as Sp1 may participate in the basic expression of phb2, while the existence of some other transcription factor binding sites and the expression differences of transcription factors in tumors or other diseases may be the main cause of PHB2 expression alteration. Nevertheless, the mechanisms of PHB2 upregulation in AP and CRC are worth to be further elucidated.

The mammalian OXPHOS system consists of five multiprotein complexes (complex I-V) and two mobile electron carriers (ubiquinone and cytochrome C) [38]. The structural and functional stability of the OXPHOS system depends on the correct synthesis, transportation and assembly of mitochondrial proteins encoded by the nuclear and mitochondrial genomes [24]. It has been shown that PHB2 affects the assembly and activity of the OXPHOS system, possibly by binding to the OXPHOS system subunits to protect them from proteolysis or by binding to the m-AAA protease to inhibit the enzyme effect [24]. However, in this study, we proposed a novel mechanism by which PHB2 affects the stability and activity of the OXPHOS system, that is, PHB2 directly interacted with NDUFS1 to enhance the binding stability of NDUFS1 to NDUFV1, which is another core subunit of complex I, thereby stabilizing OXPHOS system and promoting its activity.

As previously described, PHB2 is involved in maintaining the stability of mitochondrial structure and function [22, 24]. It has been shown that the loss of PHB2 changes the morphology of mitochondria, leading to fragmented and disorganized mitochondria, and thus induces apoptosis [24, 39, 40]. However, in this study, the knockdown of PHB2 did not induce apoptosis of CRC cells (data not shown). Considering that the possible damage of mitochondria and oxidative stress induced by PHB2 knockdown have an effect on cell proliferation, we overexpressed PHB2 and inhibited the activity of complex I with low-dose complex I inhibitors in the rescuing experiments to determine whether the increased expression of PHB2 promoted the CRC cell proliferation by upregulating the activity of mitochondrial complex I. The results indicated that partial inhibition of complex I activity reversed the increased proliferation of NCM460 and SW620 cells induced by PHB2 overexpression, suggesting that elevated PHB2 expression in CRC promotes the cell proliferation and tumorigenesis of CRC by upregulating complex I activity.

ROS is a general term for a class of chemically active molecules or ions with high oxidation activity. Cancer cells exhibit persistently elevated ROS levels due to genetic, metabolic, and microenvironment-related changes [41]. The moderately increased ROS levels are oncogenic, resulting in the activation of pro-survival signaling pathways, loss of function of tumor suppressor genes, increased glucose metabolism, and adaptation to hypoxia [42]. However, excessive ROS also can activate anti-tumor signals and initiate oxidative stress-induced tumor cell death [42]. Tumor cells expressing higher levels of antioxidant proteins reduce elevated ROS levels and establish a new redox balance, which favors maintaining oncogenic signal activation and the anti-apoptotic ability of tumor cells [41]. Our results showed that PHB2 promoted the OXPHOS of CRC cells. Increased OXPHOS levels allow more molecular oxygen to be utilized through the OXPHOS pathway, which cause the augmentation of incomplete reduced molecular oxygen ROS in CRC cells, facilitating the initiation and progression of CRC by activating tumorigenic signaling pathways. This study hints there is a potential novel mechanism by which PHB2 exerts its carcinogenic effects, but further experiments are needed to shed light on it.

## Supplementary information

Supplemental material

Original Data File

aj-checklist

## Data Availability

The datasets generated during and/or analyzed during the current study are available from the corresponding author on reasonable request.
