# Supplementary material for: PHB2 promotes colorectal cancer cell proliferation and tumorigenesis through NDUFS1-mediated oxidative phosphorylation
Source: Cell Death Dis. 2023 Jan 20;14(1):44. doi: 10.1038/s41419-023-05575-9 (PMC9852476; doi:10.1038/s41419-023-05575-9)
Supplement: Supplementary file 1 — Supplemental material [file 41419_2023_5575_MOESM1_ESM.pdf]

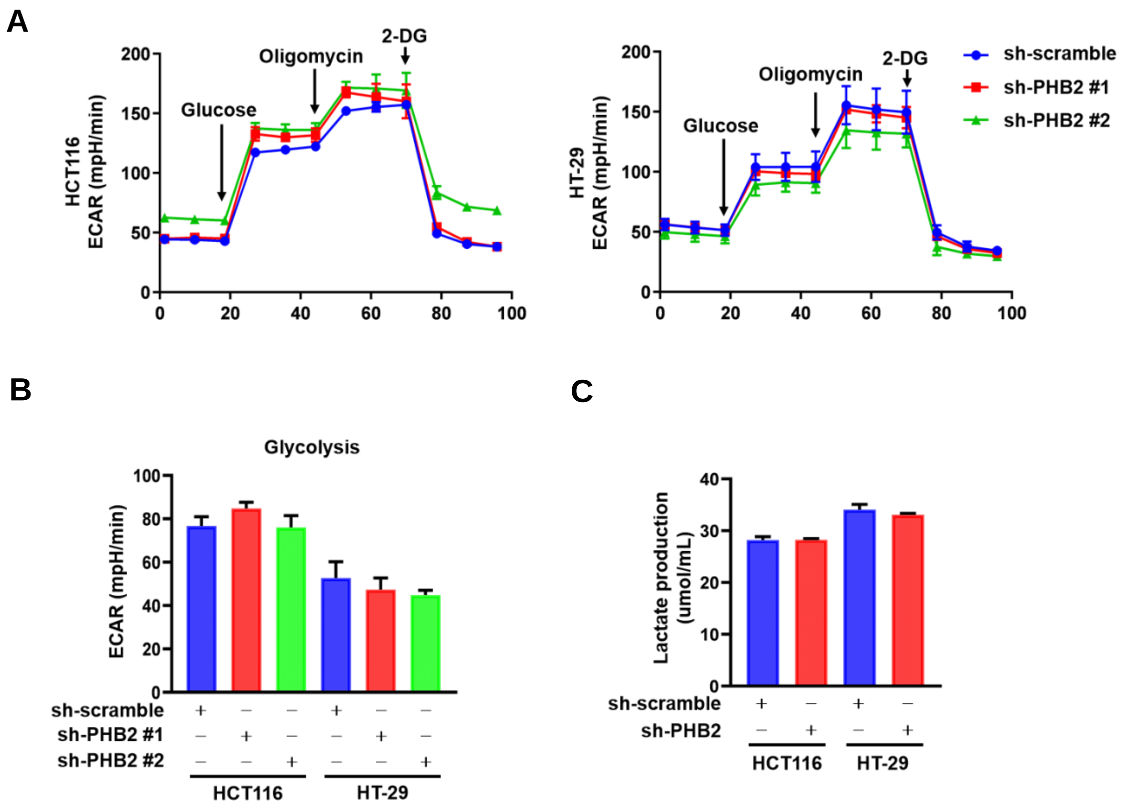

Supplementary Figure S1. PHB2 knockdown did not change the glycolysis level of colorectal cancer cells.

A Glycolytic levels in control, PHB2-deficient HCT116 and HT-29 cells were measured by Seahorse XF96 analyzer. After establishing a baseline, glucose (10 mM), oligomycin (1 μM), and 2-DG (50 mM) were sequentially added, as indicated by arrows. **B** ECAR after glucose injection indicates the glycolysis. **C** Lactic acid production in HCT116 and HT-29 cells infected with shRNA against PHB2 was indicated by absorbance measured at 570nm. Mean ± SEM.

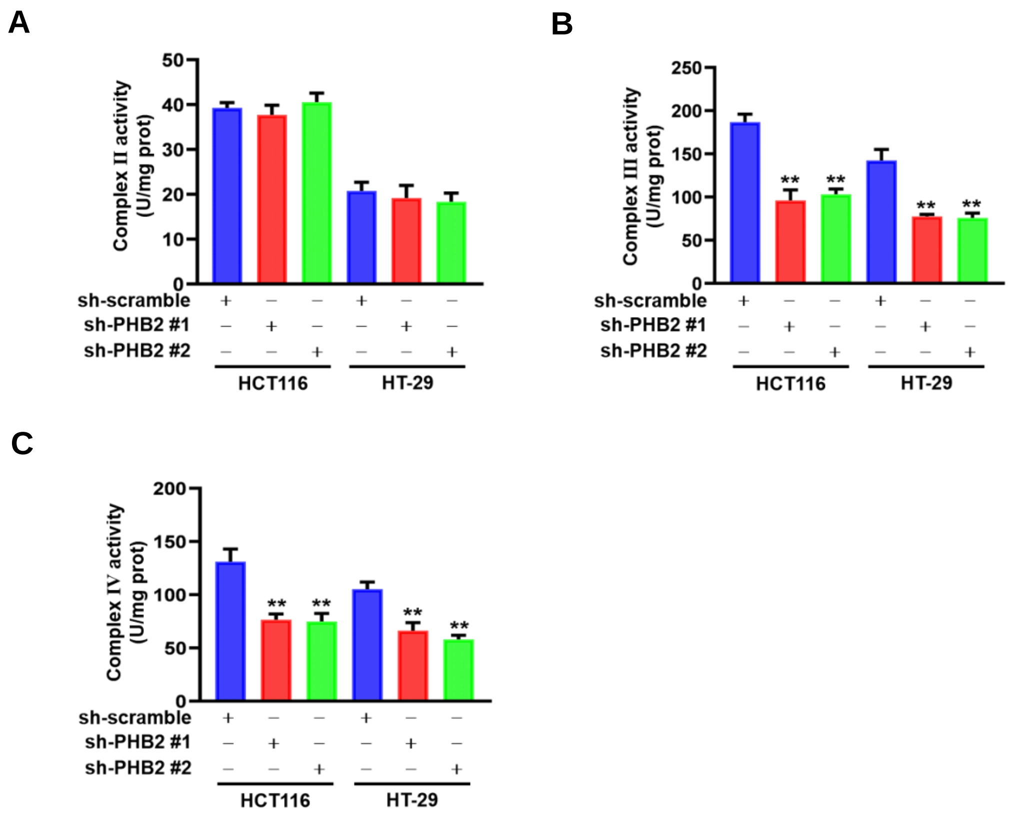

Supplementary Figure S2. Effect of PHB2 knockdown on the activity of mitochondrial respiratory chain complexes in colorectal cancer cells.

A Complex Ⅱ activity of HCT116 and HT-29 cells infected with shRNA against PHB2 was measured by a microplate reader displaying absorbance change at 605 nm for 2 min. **B** Complex Ⅲ activity was indicated by absorbance change measured at 550 nm for 2 min. **C** Complex Ⅳ activity was indicated by absorbance change measured at 550 nm for 1 min. Mean ± SEM, *p < 0.05, **p < 0.01.

Supplementary table S1. IP and LC-MS analysis of PHB2-binding proteins associated with OXPHOS in HCT116 cells

Gene name	Protein name	Unique peptides	MS score
NDUFS1	Complex I-75kD	4	140.22
UQCRC2	Ubiquinol-cytochrome c reductase core protein 2	3	107.42
ND5	NADH-ubiquinone oxidoreductase chain 5	1	22.57

Supplementary table S2 Colorectal cancer tissues

Characteristic	No. (%)
**Gender,n(%)**	
Male	21(58.33)
Female	15(41.67)
**Age (years)**	
Mean(range)	61.89(26-81)
**Stage,n(%)**	
Precancerous lesions	14(38.89)
Ⅰ-Ⅱ	11(30.56)
Ⅲ-Ⅳ	11(30.56)
