# Supplementary material for: PHB2 promotes colorectal cancer cell proliferation and tumorigenesis through NDUFS1-mediated oxidative phosphorylation
Source: Cell Death Dis. 2023 Jan 20;14(1):44. doi: 10.1038/s41419-023-05575-9 (PMC9852476; doi:10.1038/s41419-023-05575-9)

Figure 1E

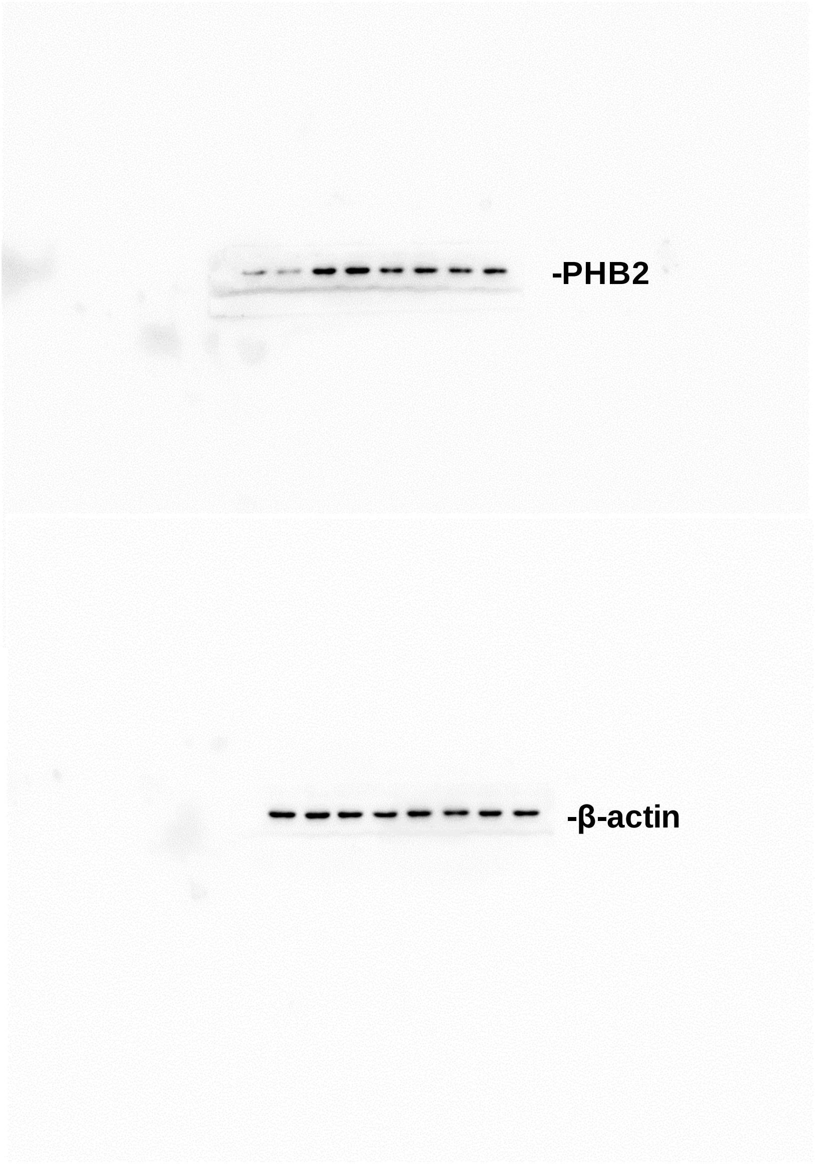

Figure 1F

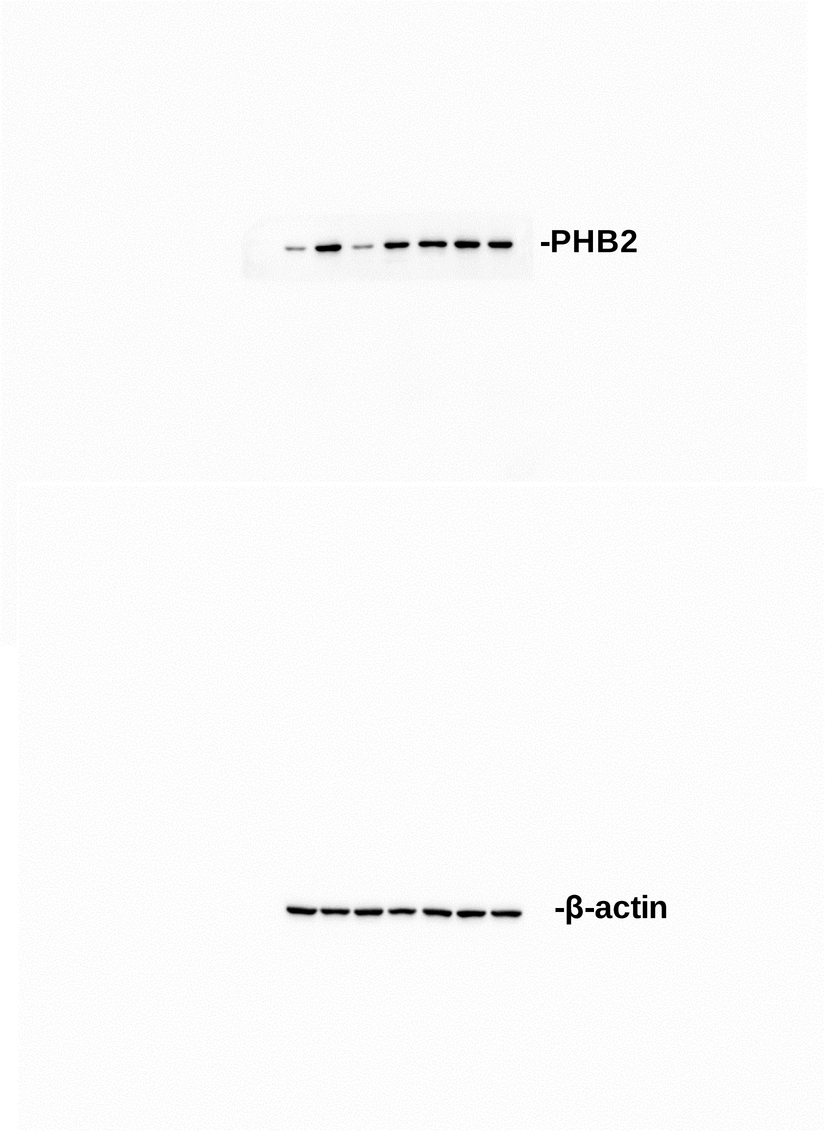

Figure 2A

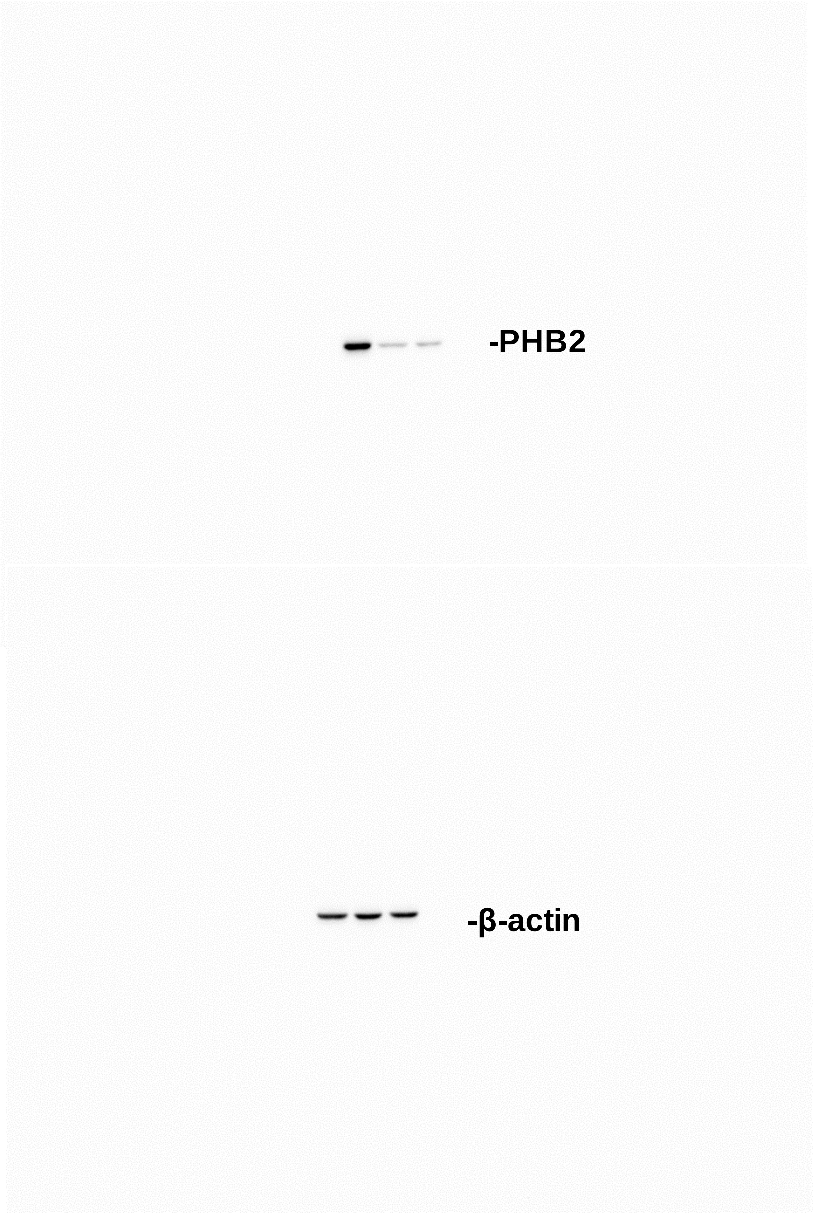

HCT116

Figure 2A

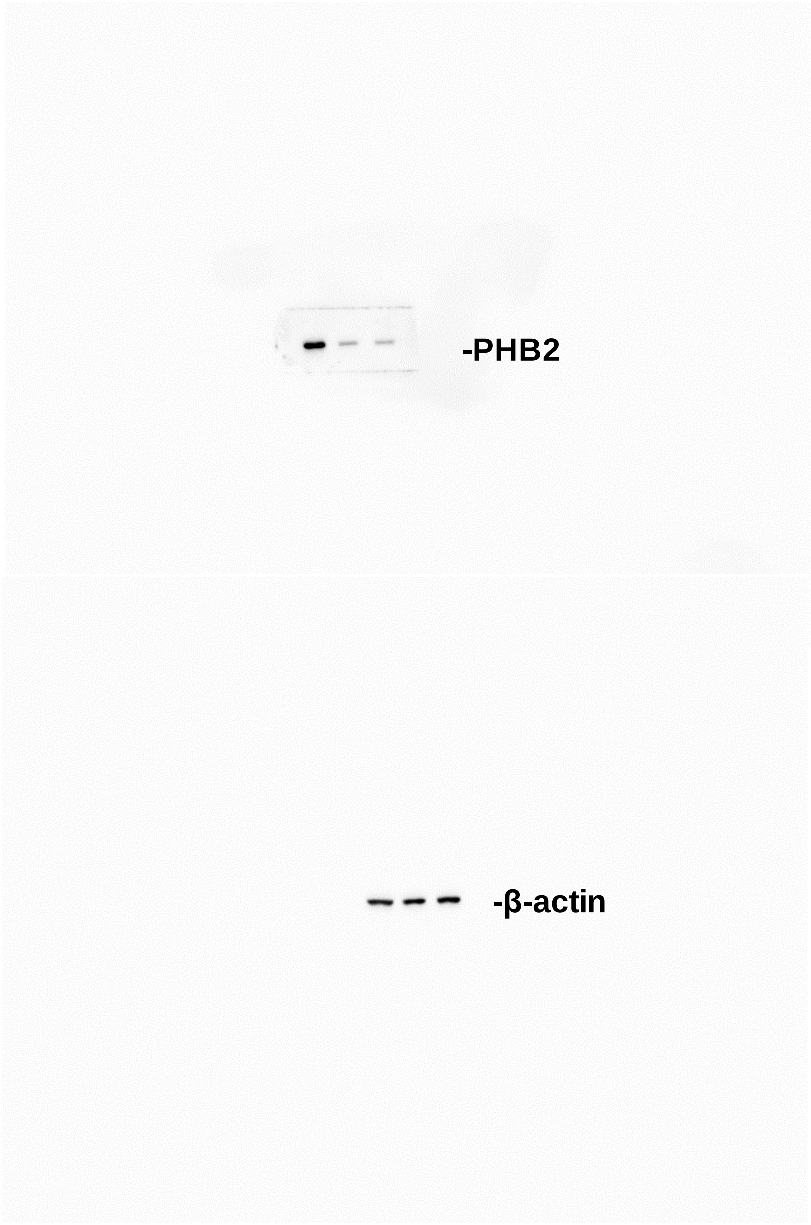

HT29

Figure 2E

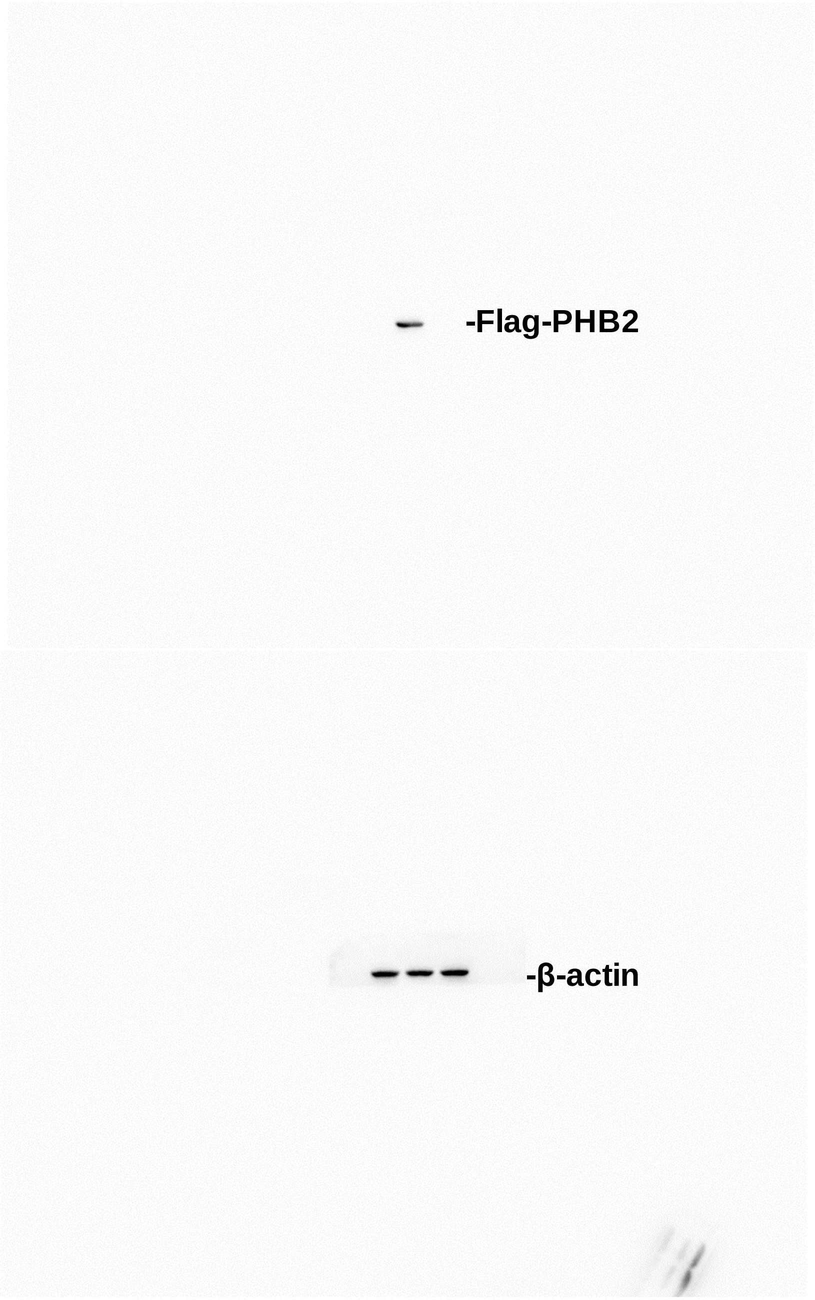

SW620

Figure 2E

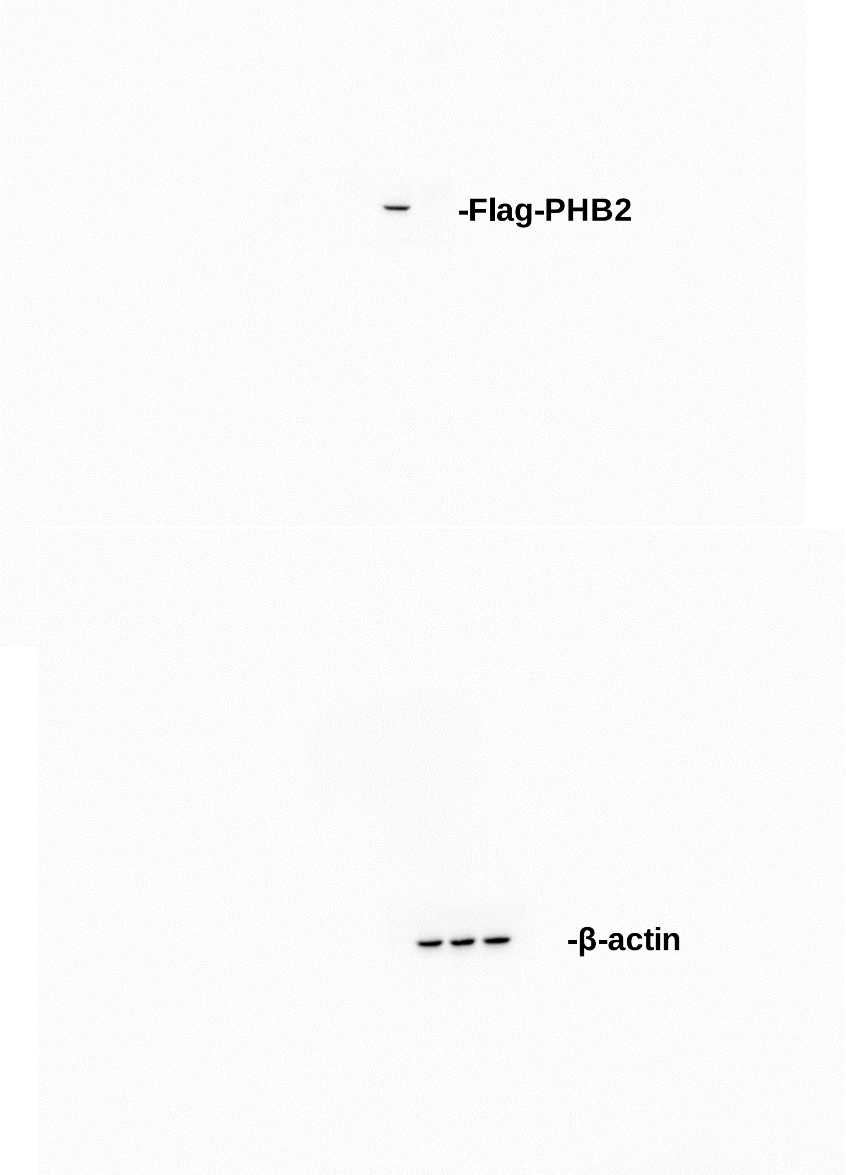

NCM460

Figure 3D

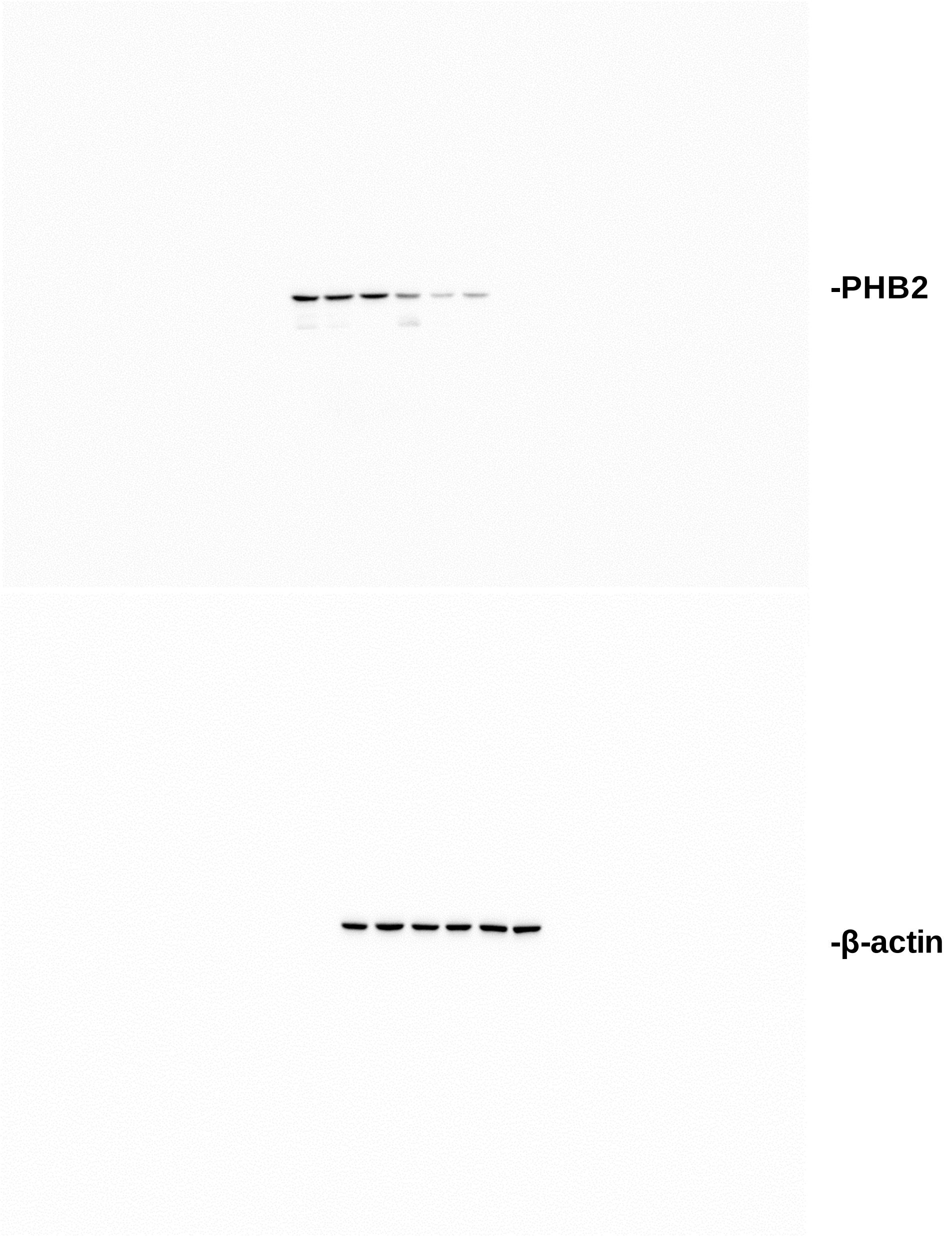

Figure 5A

HCT116 **Anti-NDUFS1**

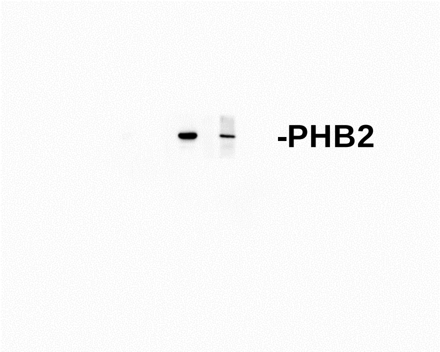

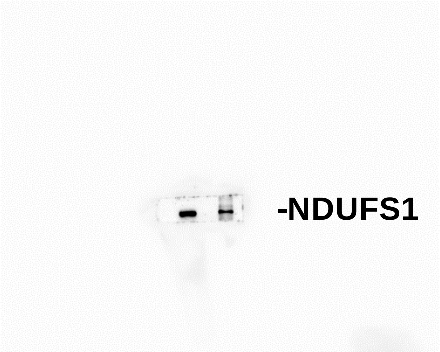

HCT116 **Anti-PHB2**

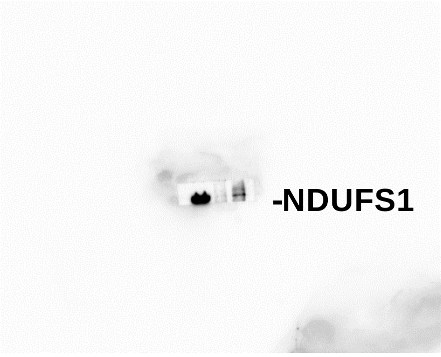

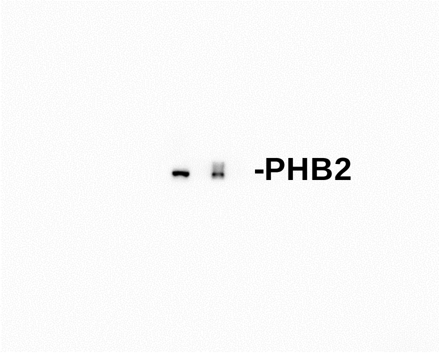

Figure 5A

HT29 Anti-NDUFS1

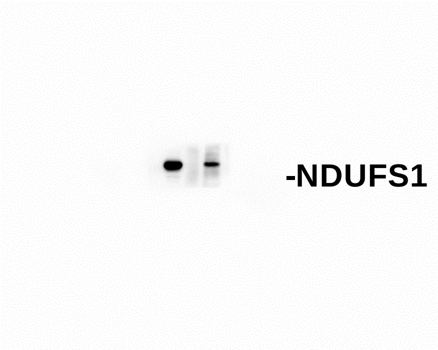

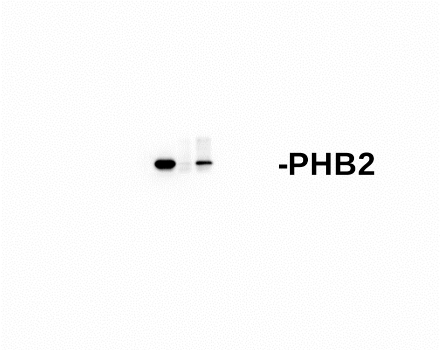

HT29 Anti-PHB2

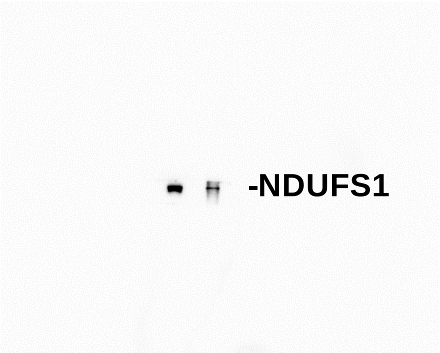

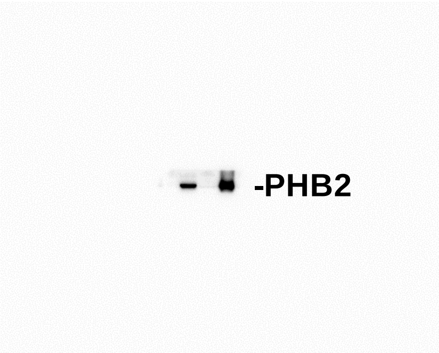

Figure 5B

Anti-Flag

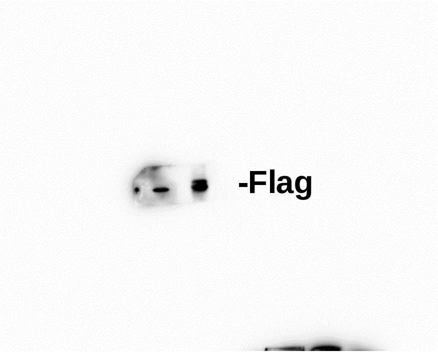

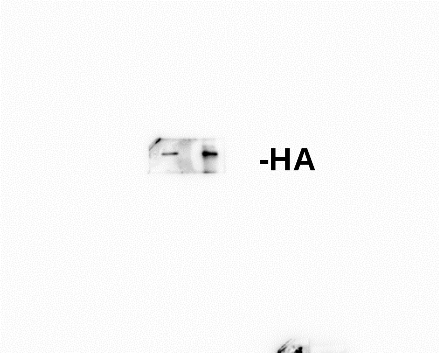

Anti-HA

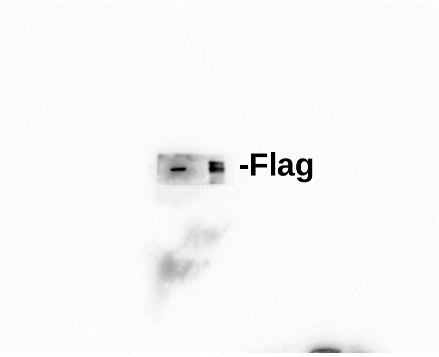

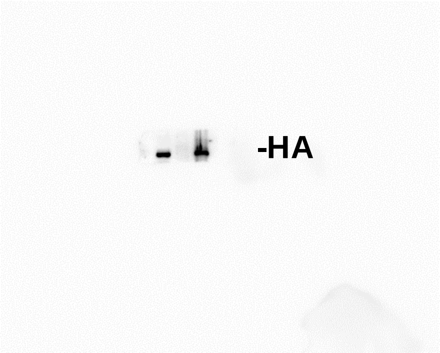

Figure 5C

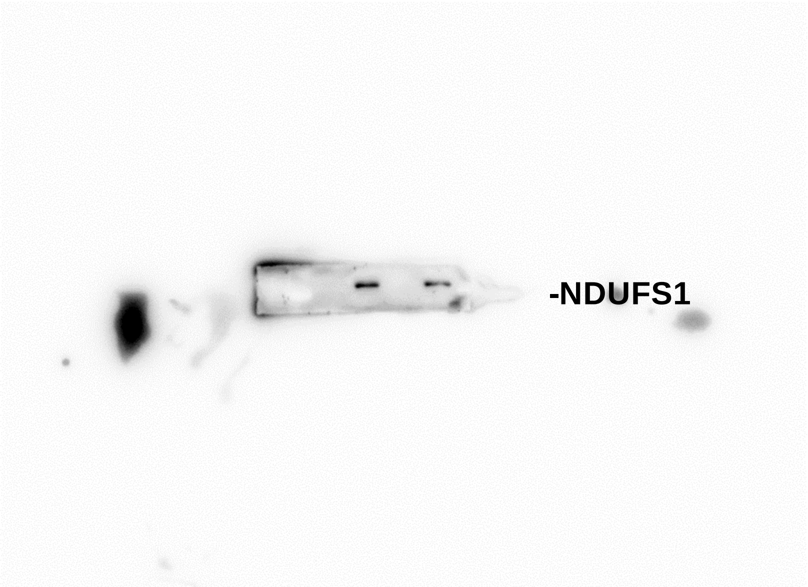

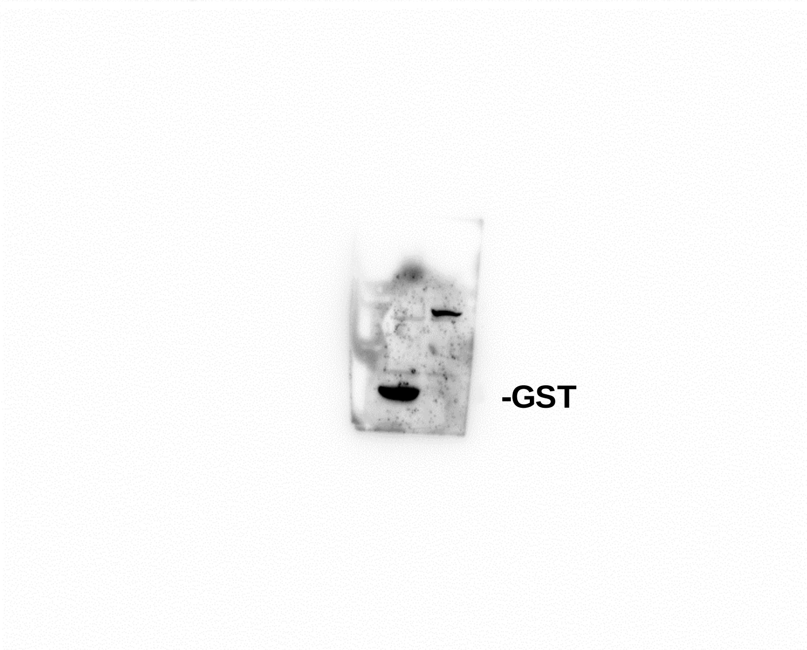

Figure 5E

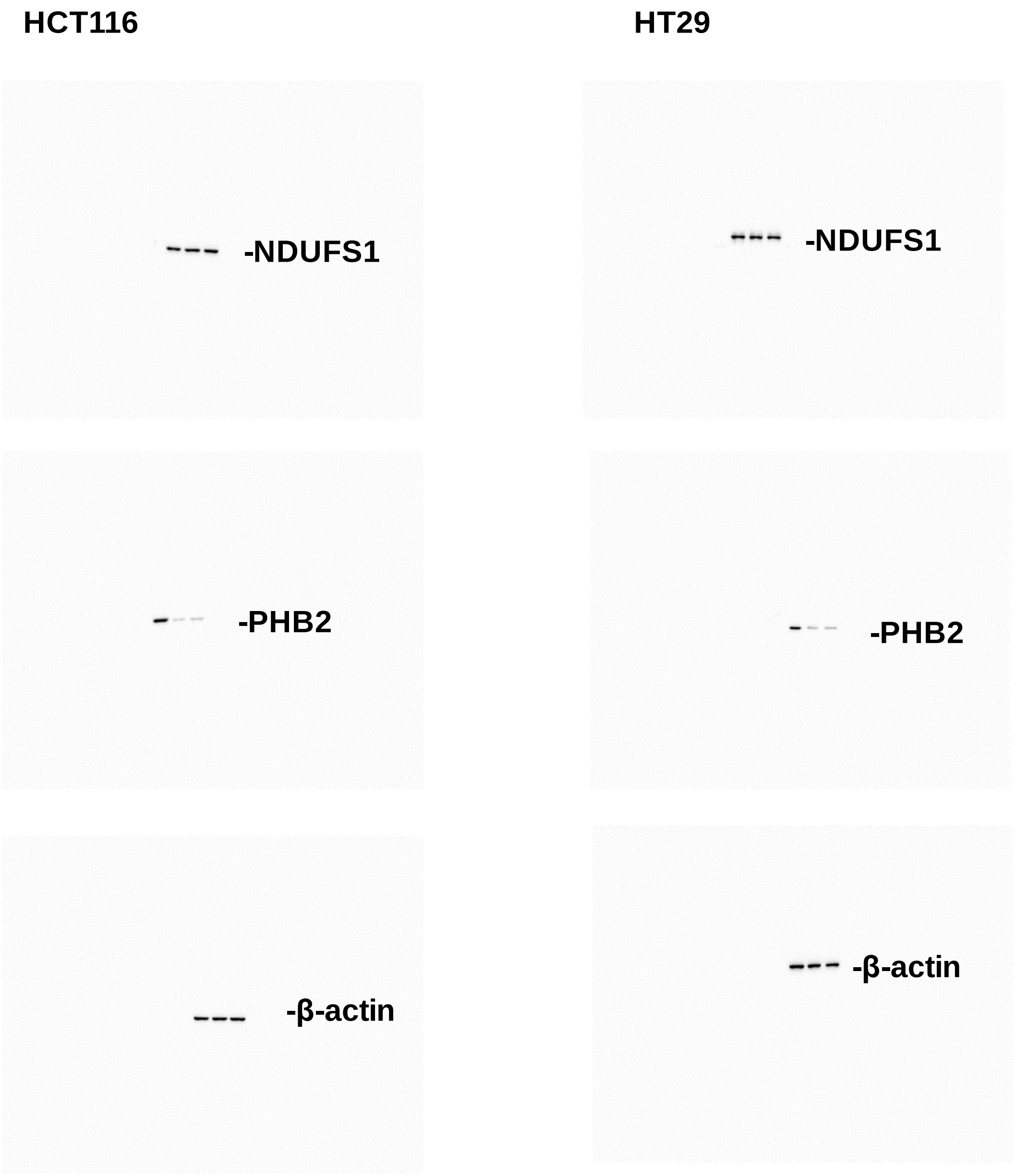

Figure 6B

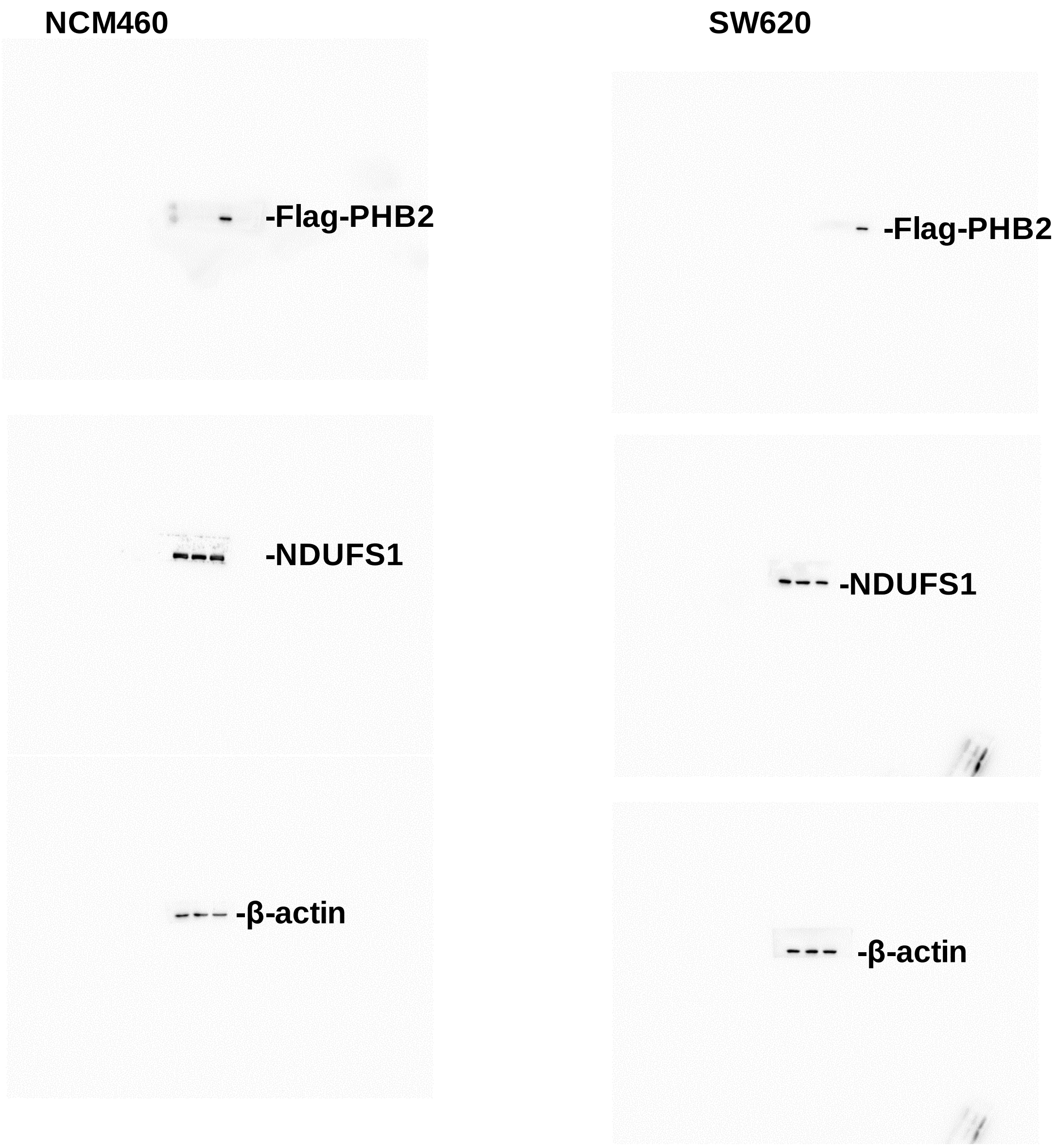
NCM460
SW620
-Flag-PHB2
-Flag-PHB2
-NDUFS1
-NDUFS1
-β-actin
-β-actin

Figure 6F

HCT116

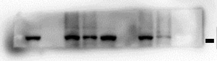
-NDUFS1

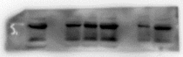
-NDUFV1

HT29

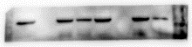
-NDUFS1

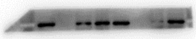
-NDUFV1

Figure 6G

HCT116 **HT29**

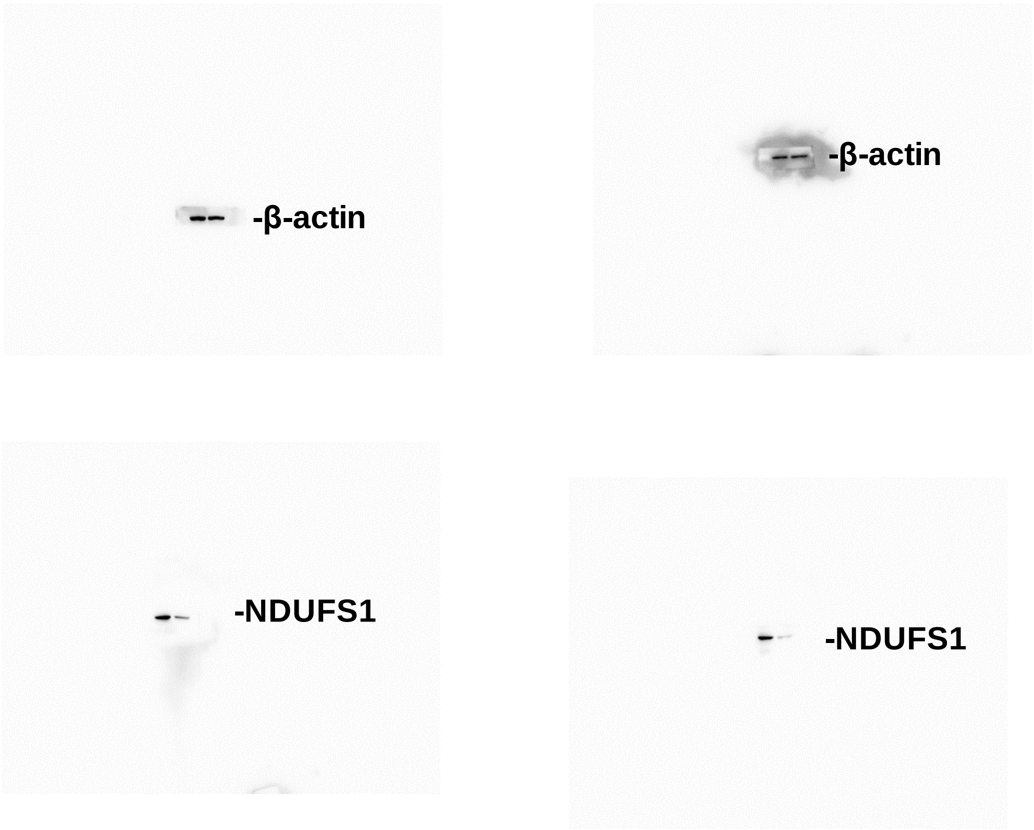

Figure 6H

HCT116 Anti-NDUFV1 siCtrl

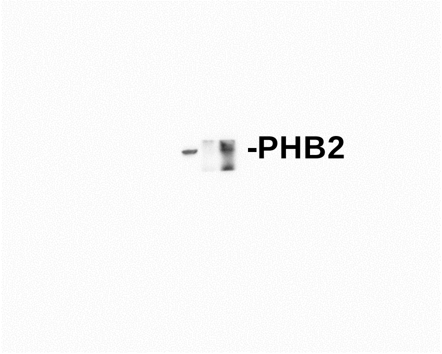

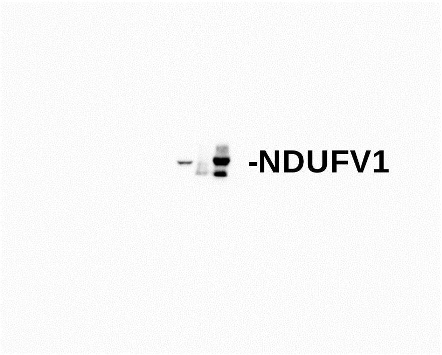

HCT116 Anti-NDUFV1 siNDUFS1

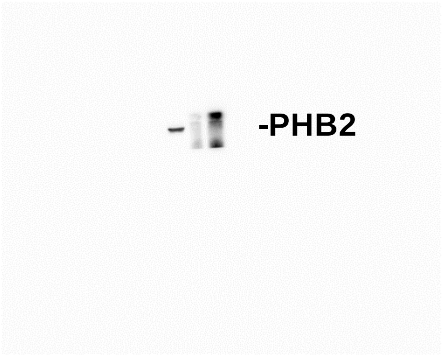

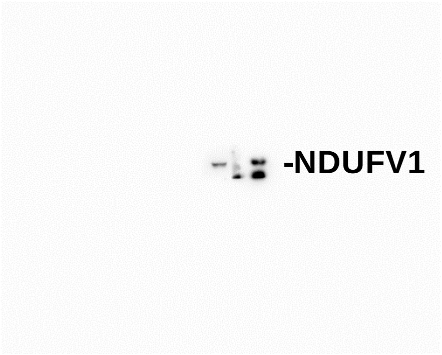

Figure 6H

HT29 Anti-NDUFV1 siCtrl

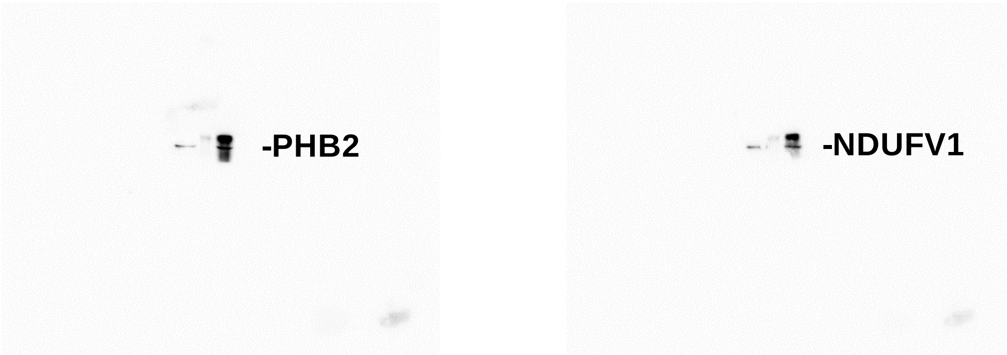

HT29 Anti-NDUFV1 siNDUFS1

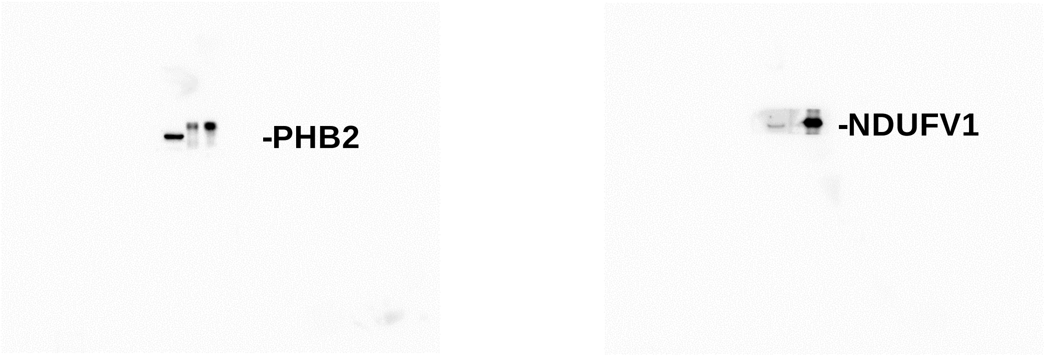

Figure 6H

HCT116 Anti-PHB2 siCtrl

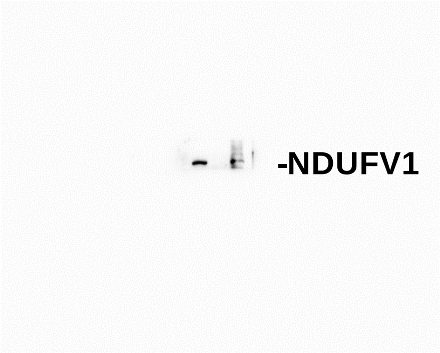

-NDUFV1

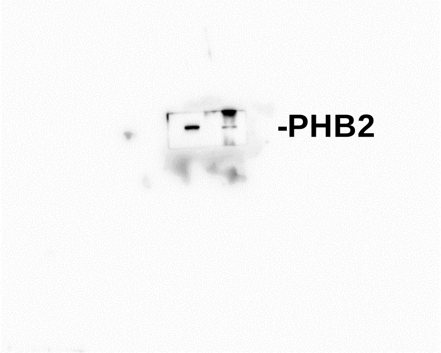

-PHB2

HCT116 Anti-PHB2 siNDUFS1

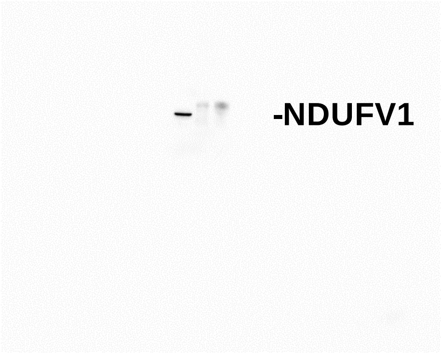

-NDUFV1

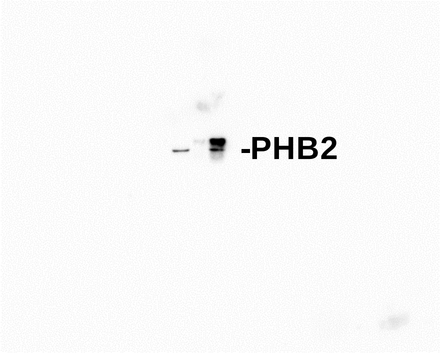

-PHB2

Figure 6H

HT29 Anti-PHB2 siCtrl

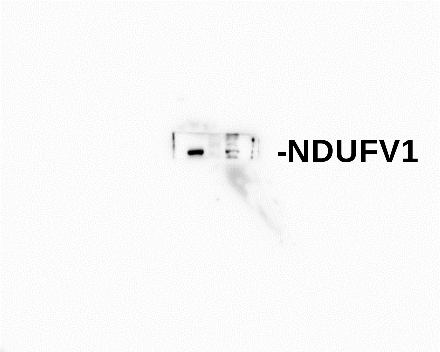

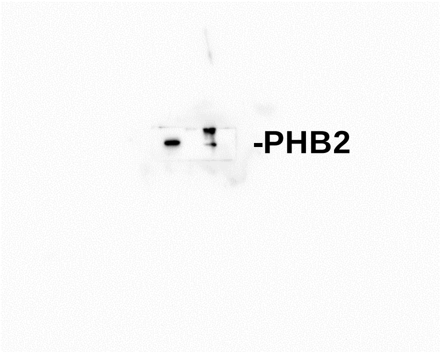

HT29 Anti-PHB2 siNDUFS1

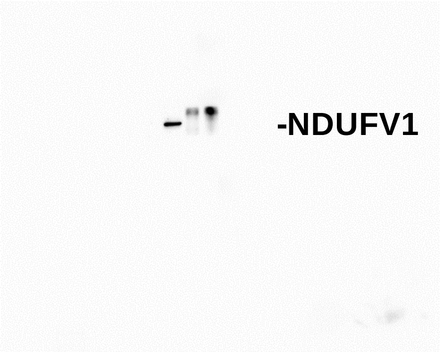

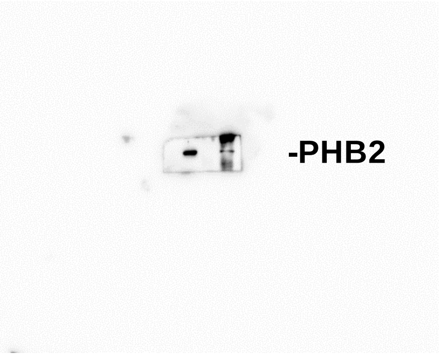

Supplement: Supplementary file 2 — Original Data File [file 41419_2023_5575_MOESM2_ESM.pdf]
